# Bone Marrow-Derived Mesenchymal Stem Cells Alleviate Cutaneous Leishmaniasis by Promoting M2 Macrophage Polarization and Skin Tissue Repair in a Murine Model

**DOI:** 10.3390/biom16060897

**Published:** 2026-06-17

**Authors:** Shirui Bai, Tao Lin, Haoxia Li, Bo Han, John P. Kastelic, Tao Zhang, Hao Shi, Gang Liu, Yipeng Jin

**Affiliations:** 1 State Key Laboratory of Veterinary Public Health and Safety, Department of Clinical Veterinary Medicine, College of Veterinary Medicine, China Agricultural University, Beijing 100193, China; shiruibai@cau.edu.cn (S.B.); s20253051246@cau.edu.cn (T.L.); llllhx@cau.edu.cn (H.L.); hanbo@cau.edu.cn (B.H.); tao.zhang@cau.edu.cn (T.Z.); haoshi@cau.edu.cn (H.S.); 2 Faculty of Veterinary Medicine, University of Calgary, Calgary, AB T2N 4N1, Canada; jpkastel@ucalgary.ca

**Keywords:** *Leishmania mexicana*, host-directed therapy, macrophage immunomodulation, skin wound healing, lesion pathology

## Abstract

Cutaneous leishmaniasis (CL) is the most common clinical form of leishmaniasis, characterized by persistent skin ulcers and nodules. Standard chemotherapeutic agents have substantial toxicity and do nothing to repair the damaged tissue, an unmet need that motivates the search for adjunctive strategies. Mesenchymal stem cells (MSCs) can modulate macrophage activity and support tissue regeneration, yet their role in CL has received limited attention. In this study, we tested whether bone marrow-derived MSCs (BM-MSCs) could attenuate *Leishmania mexicana*-induced inflammation and facilitate skin repair. Indirect co-culture of BM-MSCs with infected RAW264.7 macrophages shifted the macrophage phenotype from M1 toward M2, with higher IL-10 and Arg-1 expression and lower iNOS and IL-1β. In BALB/c mice with established CL, three weekly intravenous injections of BM-MSCs reduced paw swelling, improved skin histology, decreased type I collagen deposition, lowered Integrin β1 and Cytokeratin 17 expression, and reduced tissue parasite load. Immunofluorescence confirmed a predominantly M2 macrophage distribution in treated lesions. We inferred that BM-MSCs acted on both the immune and reparative aspects of the disease process, supporting their potential as an adjunct to conventional anti-leishmanial therapy.

## 1. Introduction

Leishmaniasis is a zoonotic disease caused by intracellular protozoa of the genus *Leishmania* and transmitted through the bites of infected phlebotomine sandflies. It ranks among the most neglected tropical diseases worldwide, placing an estimated 350 million people at risk across 98 countries and generating over 1.2 million new cases each year [1,2]. The cutaneous form (CL) accounts for approximately 70% of all cases and represents the most common clinical manifestation, with an estimated 600,000–1,000,000 new cases occurring annually worldwide [1]. CL is defined clinically by persistent skin ulcers and nodular lesions that can persist for months to years; in severe presentations, tissue destruction leads to disfigurement and lasting functional impairment [2]. Canine leishmaniasis also deserves mention, as dogs develop systemic disease with prominent dermatological involvement, and they are a primary reservoir for human infection, creating an important veterinary and public-health burden [3]. Notably, canine leishmaniasis has re-emerged in Beijing, China, with approximately 5% of dogs in mountainous areas reported to be infected [4].

Chemotherapy for leishmaniasis has undergone only limited changes over the past half-century, and each available agent has notable drawbacks. Pentavalent antimonials remain the first-line choice in many settings, but cardiac and pancreatic toxicity are common, and resistance is now widespread on the Indian subcontinent [5]. Liposomal amphotericin B achieves high cure rates yet is nephrotoxic and too expensive for most endemic countries [6,7]. Miltefosine, the only oral option, is teratogenic and is already facing emerging resistance [8,9]. Physical modalities—cryotherapy, radiofrequency heat, and CO_2_ laser—have shown benefits in selected patients but are not uniformly effective [10,11,12,13]. Perhaps most importantly, all of these approaches target the parasite but leave the lesion itself largely unaddressed; patients may achieve a parasitological cure but still have non-healing ulcers that are prone to secondary infection. A treatment that could provide both infection control and tissue repair would therefore fill an important gap.

Mesenchymal stem cells (MSCs) have attracted interest in inflammatory and immune-mediated diseases because of their capacity to secrete paracrine factors that reshape local immune responses and support tissue regeneration [14]. In CL, the macrophage sits at the intersection of parasite biology and host pathology; it is the primary intracellular host for *Leishmania* amastigotes and a central driver of inflammation. M1-polarized macrophages produce iNOS-derived NO, IL-1β, and TNF-α that can kill parasites, whereas M2-polarized macrophages secrete IL-10 and arginase-1 (Arg-1) and promote wound healing but may also create a more permissive environment for parasite persistence [15,16]. Whether and how MSCs might recalibrate this balance in the setting of active *Leishmania* infection are important questions, with both mechanistic and practical relevance.

Prior work has produced mixed results. In co-culture studies, MSCs reduced macrophage inflammatory activity after *Leishmania* stimulation [17,18], and murine experiments with adipose- or bone marrow-derived MSCs reported variable changes in lesion size, parasite burden, and Th1/Th2 cytokine profiles [19,20,21]. In one *L. amazonensis* model, MSC administration actually increased parasite load and IL-10 levels [21]; in another, combining MSCs with meglumine antimoniate reduced both lesion size and parasite numbers [22]. These discrepancies make it difficult to draw firm conclusions about MSC efficacy in CL, and the specific question of whether BM-MSCs can promote skin repair concurrent with infection control has not been directly addressed.

In this study, we used *L. mexicana*-infected BALB/c mice—a susceptible strain that develops chronic, ulcerative lesions—together with complementary in vitro macrophage co-culture models to determine if BM-MSC treatment could shift macrophage polarization toward M2, reduce inflammatory gene expression, improve skin pathology, and decrease tissue parasite burden. The findings provide new knowledge on the feasibility of MSCs as an adjunctive intervention in CL and on mechanisms through which they might act.

## 2. Materials and Methods

### 2.1. Experimental Animals and Ethics Statement

Six- to eight-week-old female BALB/c mice were purchased from Spefo (Beijing) Biotechnology Co., Ltd. (Beijing, China) and housed under specific pathogen-free conditions at the Laboratory Animal Center of China Agricultural University. Standard rodent chow and water were available ad libitum, and mice were acclimatized for 1 wk before any experimental procedure. All protocols were reviewed and approved by the Institutional Animal Care and Use Committee of the College of Veterinary Medicine, China Agricultural University (Approval No. AW02113202-2-2).

### 2.2. Parasites and Cell Lines

*Leishmania mexicana* promastigotes (strain MNYC/BZ/62/M379) were a kind gift from Beijing Friendship Hospital. After rapid thawing, parasites were pelleted at 300× *g* for 10 min, resuspended in Schneider’s insect medium (Sigma-Aldrich, St. Louis, MO, USA) with 10% heat-inactivated FBS (Gibco, Thermo Fisher Scientific, Waltham, MA, USA), and maintained at 22–25 °C without CO_2_. Virulence was sustained by serial passage through BALB/c mice. Stationary-phase promastigotes from days 4–6 of culture were used throughout.

RAW264.7 cells were obtained from Shanghai Jinyuan Biotechnology Co., Ltd. (Shanghai, China). BM-MSCs derived from BALB/c mice were from Jingxiang Qidian Cell Technology Co., Ltd. (Shanghai, China); cells at passages 3–5 were used for the experiments, and epidermal stem cells were from Yi’aobang Biotechnology Research Co., Ltd. (Beijing, China). All mammalian cells were maintained in DMEM (Gibco) with 10% FBS and 1% penicillin–streptomycin (Gibco) at 37 °C, 5% CO_2_.

### 2.3. In Vitro Infection and Indirect Co-Culture Model

RAW264.7 macrophages were infected with stationary-phase promastigotes at a 10:1 parasite-to-cell ratio and incubated at 37 °C, 5% CO_2_. Cells were collected at 24, 48 and 96 h post-infection for downstream analysis. For co-culture experiments, infected or uninfected RAW264.7 cells were seeded in the lower compartment of 12-well plates. After 12 h, epidermal stem cells (2 × 10^5^ cells/mL) were placed in Transwell inserts (0.4 μm; Corning Inc., Corning, NY, USA) above the macrophages, allowing for paracrine exchange without direct contact. Three groups were run in parallel: a blank control (RAW264.7 only), a negative control (RAW264.7 + uninfected epidermal stem cells), and an infected group (*L. mexicana*-infected RAW264.7 + epidermal stem cells; *n* = 3 biological replicates). Co-cultures were maintained for 24 h before harvest.

### 2.4. Murine Model of Cutaneous Leishmaniasis

After acclimatization, mice were randomized into a naïve control group and an infected group (*L.MEX*; *n* = 10 per group). The *L.MEX* mice received a subcutaneous injection of 1 × 10^6^ stationary-phase promastigotes in 50 μL sterile PBS into the right hind paw pad; controls received PBS alone. Body weight, feed and water consumption, and the appearance of the inoculation site were recorded weekly for 12 wk. At Week 12, five animals per group were euthanized under isoflurane, and paw pad tissue, adjacent skin and spleens were harvested aseptically.

### 2.5. Macrophage Polarization Assay and MSC Co-Culture

RAW264.7 cells were polarized to M1 by treatment with LPS (100 ng/mL; Sigma-Aldrich) and IFN-γ (20 ng/mL; PeproTech, Cranbury, NJ, USA) for 24 h, or to M2 by IL-4 (20 ng/mL; PeproTech) for 24 h. Polarized macrophages were then placed beneath BM-MSCs (2 × 10^5^ cells/mL) seeded in 0.4 μm Transwell inserts for 48–96 h. The insert design restricted communication to secreted factors.

### 2.6. Flow Cytometry

Cells were harvested, washed twice in ice-cold PBS and stained for 30 min at 4 °C with fluorochrome-conjugated antibodies against CD86 (M1 marker) and CD206 (M2 marker). After washing, cells were passed through a 40 μm strainer and immediately acquired. Data were analyzed in FlowJo v10.8.1 (BD Biosciences, Franklin Lakes, NJ, USA).

### 2.7. In Vivo BM-MSC Treatment

At Week 12 post-infection, mice were redistributed into three groups (*n* = 6 per group): naïve control, untreated infected (*L.MEX*), and BM-MSC-treated infected (*L.MEX* + MSC). Treated mice received tail-vein injections of 1 × 10^6^ BM-MSCs in 200 μL PBS once weekly for three consecutive weeks; the other groups received PBS on the same schedule. All animals were euthanized 28 d after the last injection, and skin lesion tissues were collected for histology, immunofluorescence, molecular analysis, and parasite quantification.

### 2.8. RNA Extraction and RT-qPCR

Total RNA was extracted from cells, and tissue was snap-frozen using a commercial kit (Foregene, Chengdu, China), according to the manufacturer’s instructions. Concentration and purity were checked by spectrophotometry (A260/A280 ≥ 1.8), and integrity was confirmed by agarose gel electrophoresis. One microgram of RNA was reverse-transcribed with a commercial kit (Vazyme, Nanjing, China), and qPCR was run in a SYBR Green system (Vazyme) on an Applied Biosystems instrument (Waltham, MA, USA). Each sample was measured in technical triplicate alongside a no-template control. Relative expression was calculated by the 2^−ΔΔCT^ method, using β-actin as the reference. Primers are in Table 1.

### 2.9. Western Blot Analysis

Cells and minced tissue were lysed in RIPA buffer with 1 mM PMSF and a protease inhibitor cocktail (Beyotime, Shanghai, China) and then centrifuged at 12,000× *g*, 4 °C for 15 min. Protein concentration was measured by BCA assay. Equal amounts of protein were separated by SDS-PAGE, transferred to PVDF membranes, and blocked in 5% non-fat milk (TBST, 1 h, room temperature). Membranes were incubated overnight at 4 °C with primary antibodies against iNOS, IL-1β, Arg-1, Integrin β1, and Cytokeratin 17 (Proteintech, Wuhan, China) and then with HRP-conjugated secondary antibodies for 1 h. Bands were detected by enhanced chemiluminescence and quantified with ImageJ 1.51K (NIH, Bethesda, MD, USA).

### 2.10. Histological Analysis and Immunofluorescence Staining

Skin lesion tissues were fixed in 10% neutral-buffered formalin, paraffin-embedded, and cut into 5 μm sections. H&E staining was used to assess tissue architecture and inflammatory infiltration. Under polarized light, Sirius Red staining distinguished type I collagen (thick, red-orange birefringent fibers) from type III collagen (thin, green fibers), providing a measure of fibrosis and remodeling quality.

For immunofluorescence, sections were deparaffinized, rehydrated, and subjected to heat-induced antigen retrieval, followed by blocking with 5% BSA for 30 min. After overnight incubation with primary antibodies against iNOS and Arg-1 at 4 °C, sections were incubated with Alexa Fluor-conjugated secondary antibodies for 1 h and counterstained with DAPI. Images were acquired by confocal laser scanning microscopy.

### 2.11. Parasite Burden Quantification

Genomic DNA was extracted from ~20 mg of skin lesion tissue (Tiangen, Beijing, China). Parasite numbers were determined by TaqMan qPCR targeting kinetoplast DNA (kDNA): forward 5′-TGTAAAATAGGGGCGGGTGG-3′, reverse 5′-CACCCAAAACCAAGCCCAAC-3′, probe 5′-FAM-GGCCGGAAATGGCTCCCCCTGGGCT-TAMRA-3′. A standard curve generated from serial 10-fold dilutions of promastigote DNA (10^6^–10^0^ parasites, with each dilution mixed with skin tissue collected from uninfected mice) was used to convert CT values into copy numbers, reported as parasites per milligram of tissue.

### 2.12. Statistical Analysis

Data are presented as mean ± SD from at least three independent experiments. Group comparisons were made by unpaired Student’s *t*-test (two groups) or one-way ANOVA followed by Tukey’s post hoc test (three or more groups), using GraphPad Prism v8.0.2 (San Diego, CA, USA). A *p*-value < 0.05 was considered significant (* *p* < 0.05; ** *p* < 0.01; ns, not significant).

## 3. Results

### 3.1. L. mexicana Infection Drives M1 Macrophage Polarization In Vitro

**Figure 1:**
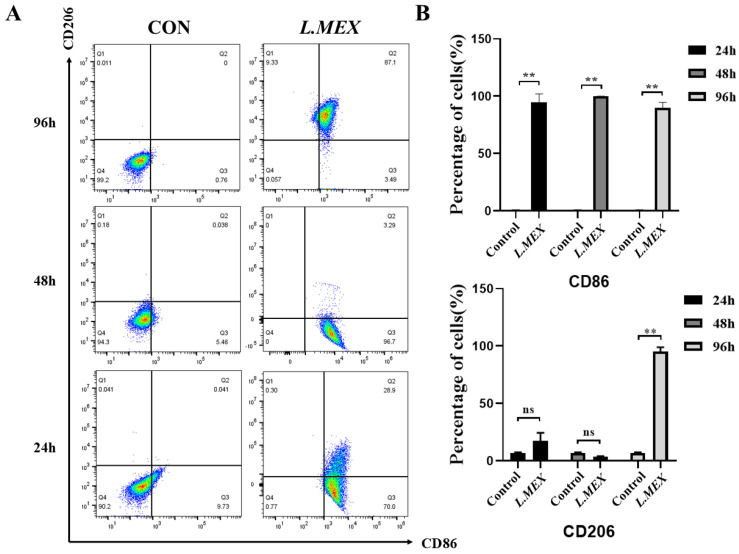
Macrophage polarization following *L. mexicana* infection. (**A**) Flow cytometry dot plots showing CD86 (M1) and CD206 (M2) surface expression on RAW264.7 cells at 24, 48, and 96 h post-infection. (**B**) Quantification of CD86^+^ and CD206^+^ cells. Data are mean ± SD, *n* = 3. ** *p* < 0.01; ns, not significant.

We first assessed how macrophage phenotype changes over the course of *L. mexicana* infection. Based on flow cytometry at 24, 48 and 96 h post-infection, CD86 (M1 marker) was strongly upregulated at all three time points (81.6, 95.9, and 92.97%, respectively) in infected cells versus corresponding controls (all *p* < 0.01; Figure 1). CD206 (M2 marker) expression remained low at 24 h (17.2%) and 48 h (3.0%), with no significant difference from uninfected cells. At 96 h, however, CD206 positivity rose sharply to 96.9% (*p* < 0.01), indicating that a late-stage mixed phenotype emerged as infection progressed.

**Figure 2:**
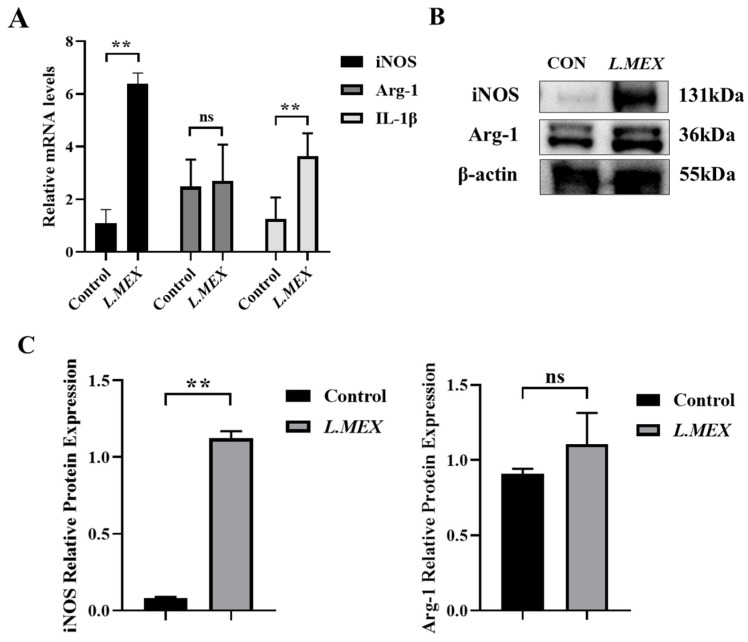
*L. mexicana* infection upregulates M1 markers in RAW264.7 macrophages. (**A**) Relative mRNA expression of IL-1β, iNOS, and Arg-1. (**B**) Representative Western blots for iNOS and Arg-1. (**C**) Densitometric quantification normalized to β-actin. RAW264.7 macrophages were infected with *L. mexicana* promastigotes and analyzed at 24 h post-infection. Data are mean ± SD, *n* = 3. ** *p* < 0.01; ns, not significant. Original Western blots images can be found in the Supplementary Materials.

Gene and protein expression analyses were consistent with flow cytometry data. iNOS mRNA and protein were significantly elevated from 24 h onward (*p* < 0.01), as was IL-1β mRNA (*p* < 0.01; Figure 2). Arg-1 mRNA and protein had no significant change (*p* > 0.05). Taken together, these data indicated that *L. mexicana* infection initially enforced an M1-associated, pro-inflammatory state, with partial acquisition of CD206-associated M2 features only at 96 h.

### 3.2. BM-MSC Co-Culture Shifts Macrophage Polarization Toward M2

**Figure 3:**
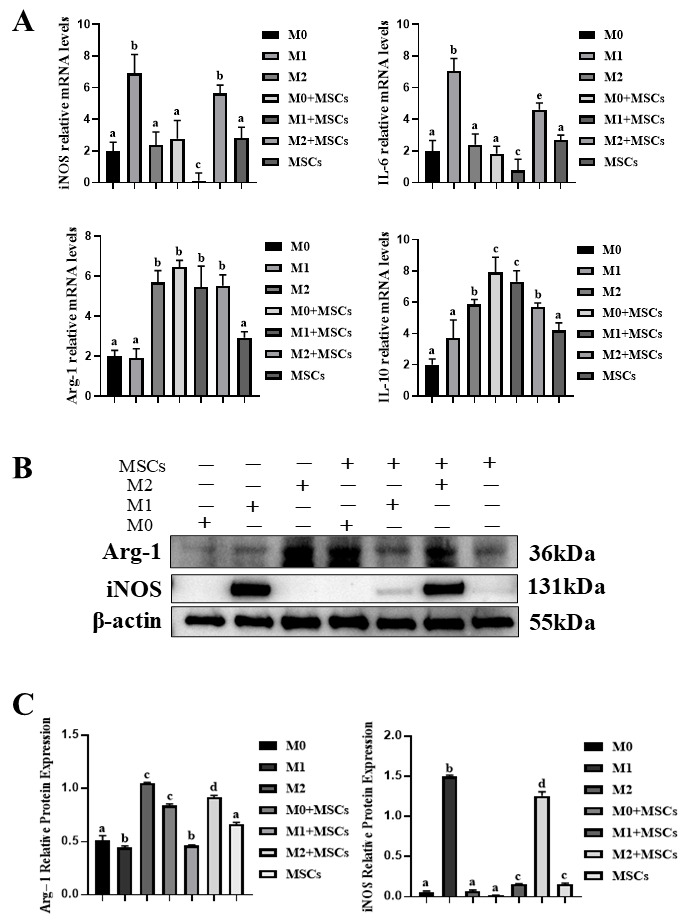
BM-MSC co-culture promotes M2 polarization in M1-stimulated macrophages. (**A**) mRNA expression of IL-10, Arg-1, and iNOS after co-culture with BM-MSCs. (**B**) Western blot for Arg-1, iNOS, and IL-10. “+” and “−” indicate the presence or absence of the indicated cell type; lanes containing both macrophages and MSCs represent Transwell co-culture conditions. (**C**) Densitometric quantification. Data are mean ± SD, *n* = 3. Different letters indicate statistically significant differences; shared letters indicate no significant difference (one-way ANOVA, Tukey’s post hoc). Original Western blots images can be found in the Supplementary Materials.

To test whether BM-MSCs could modulate macrophage polarization under different stimulation conditions, untreated M0 macrophages, LPS/IFN-γ-induced M1 macrophages, and IL-4-induced M2 macrophages were co-cultured with BM-MSCs in a Transwell system (Figure 3). In both conditions, BM-MSC co-culture increased IL-10 and Arg-1 mRNA and protein relative to the respective controls (*p* < 0.01), whereas iNOS and IL-1β expression decreased. The M2-promoting effect suggests that paracrine BM-MSC signals are sufficient to reprogram macrophage phenotype in the presence of ongoing inflammatory stimulus. Notably, BM-MSC co-culture also significantly increased iNOS expression in M2-stimulated macrophages.

### 3.3. L. mexicana Damages Epidermal Stem Cells and Induces Skin Pathology

**Figure 4:**
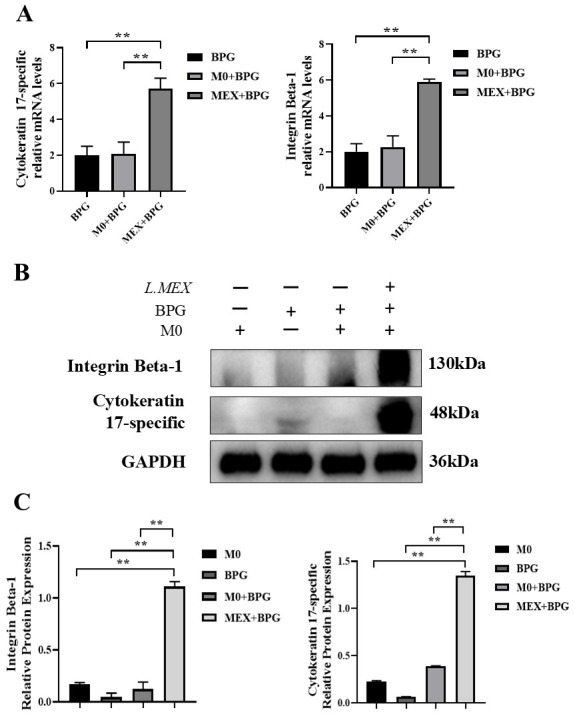
*L. mexicana*-infected macrophages damage epidermal stem cells via paracrine signaling. (**A**) Relative mRNA expression of Integrin β1 and Cytokeratin 17 in co-cultured epidermal stem cells. (**B**) Western blots for both markers. (**C**) Densitometric quantification. M0, uninfected RAW264.7; BPG, epidermal stem cells; MEX, *L. mexicana*-infected. Data are mean ± SD, *n* = 3. ** *p* < 0.01. Original Western blots images can be found in the Supplementary Materials.

Integrin β1 and Cytokeratin 17 are markers of epidermal stem cell activation associated with skin injury. In the co-culture model, infection significantly increased mRNA and protein levels of both markers in epidermal stem cells compared to uninfected controls (*p* < 0.01; Figure 4), suggesting that macrophages conditioned by *L. mexicana* release factors that damage the epidermal compartment.

**Figure 5:**
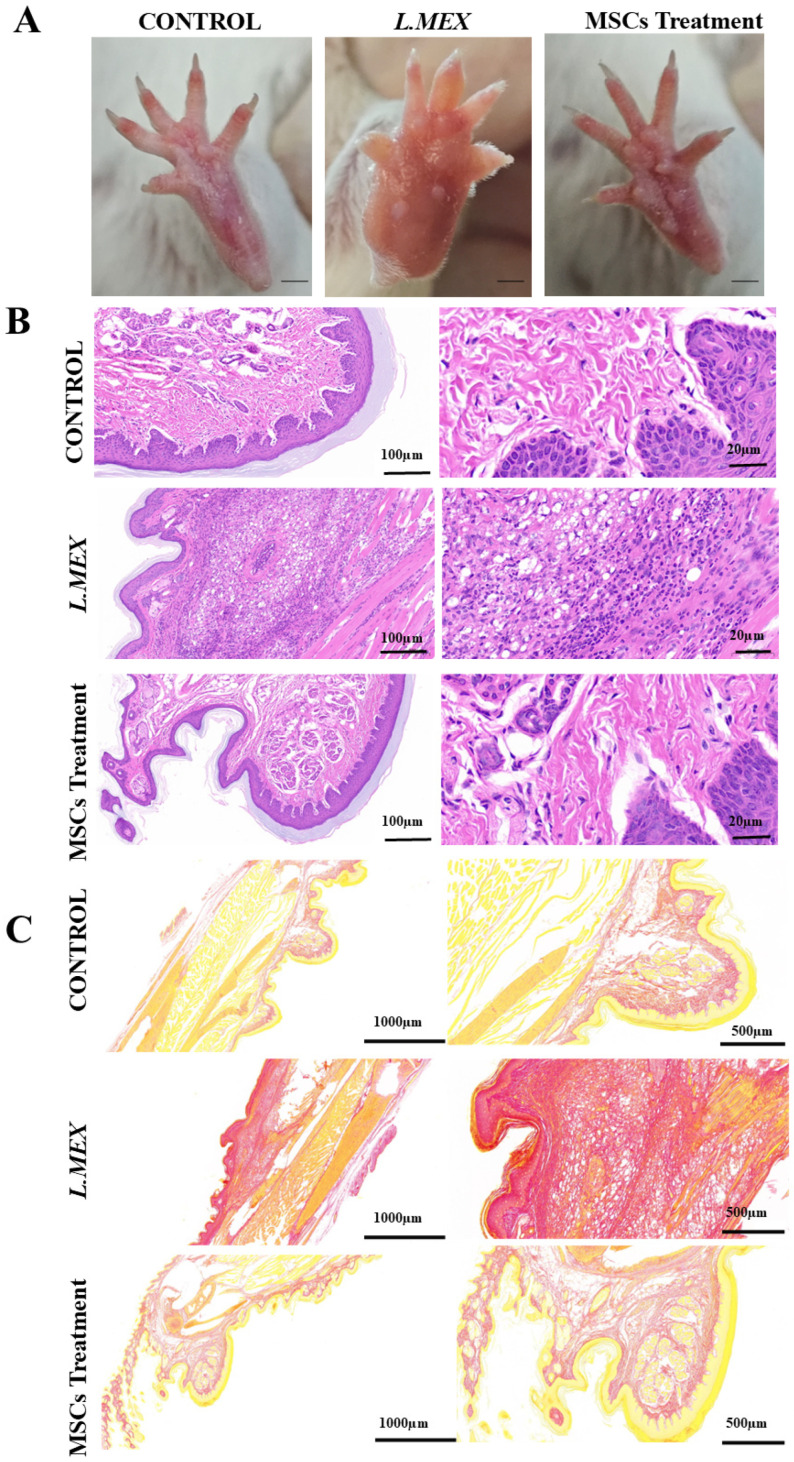
BM-MSC treatment reduces lesion severity in infected mice. (**A**) Representative photographs of paw pads at the experimental endpoint. Scale bar = 1.0 mm. (**B**) H&E-stained sections showing tissue architecture and inflammatory infiltration. (**C**) Sirius Red-stained sections; type I collagen appears red-orange and type III collagen appears green under polarized light. Magnification scales are indicated in each panel.

In BALB/c mice, visible paw swelling appeared from Week 4 and progressed through Week 12 (Figure 5A). Histologically, there was a disrupted epithelium, extensive neutrophil infiltration, and heavy inflammatory cell accumulation (Figure 5B). In addition, Sirius Red staining revealed a marked increase in type I collagen deposition, consistent with fibrotic remodeling (Figure 5C).

### 3.4. BM-MSC Treatment Reduces Lesion Severity, Parasite Burden, and M1 Marker Expression In Vivo

Mice that received three weekly BM-MSC injections beginning at Week 12 had substantially reduced paw swelling by the experimental endpoint, 28 d later (Figure 5A). Histologically, the improvement was clear; inflammatory infiltration was considerably lower, and Sirius Red staining showed a shift from type I toward type III collagen, indicating more orderly, less fibrotic tissue repair (Figure 5B,C).

**Figure 6:**
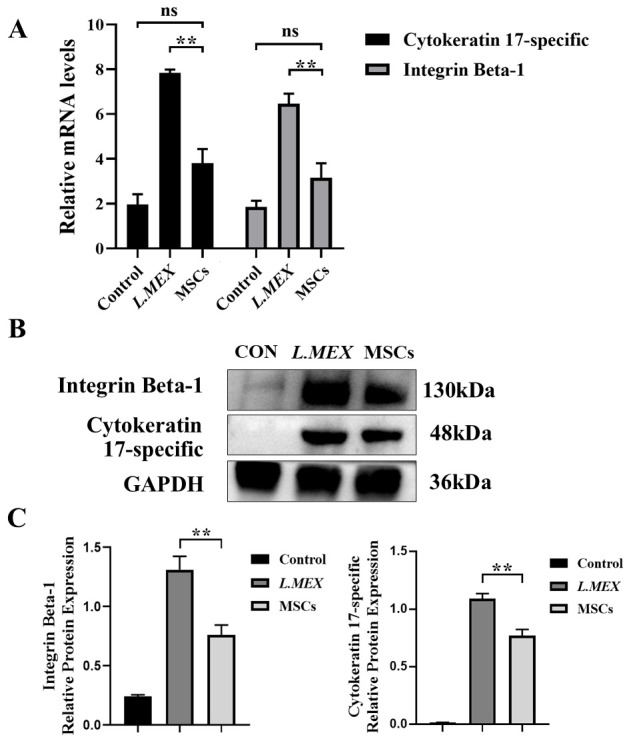
BM-MSC treatment reduces skin injury marker expression in vivo. (**A**) Relative mRNA expression of Integrin β1 and Cytokeratin 17. (**B**) Western blots. (**C**) Densitometric quantification. Data are mean ± SD, *n* = 6. ** *p* < 0.01; ns, not significant. Original Western blots images can be found in the Supplementary Materials.

**Figure 7:**
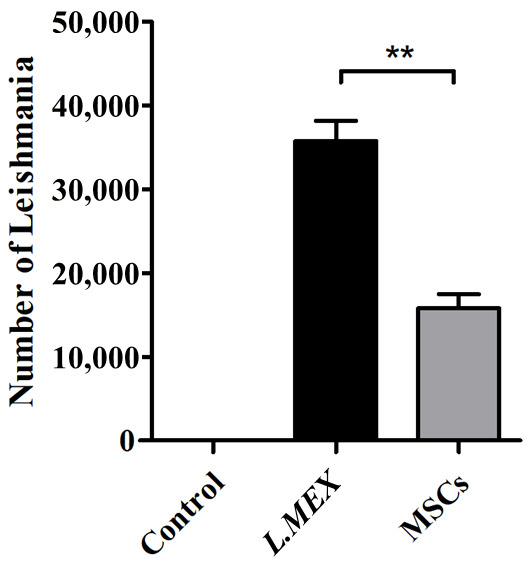
BM-MSC treatment reduces tissue parasite burden. Parasite load (copies/mg tissue) in skin lesions of untreated infected and BM-MSC-treated mice, quantified by kDNA-targeting TaqMan qPCR. Data are mean ± SD, *n* = 6. ** *p* < 0.01.

**Figure 8:**
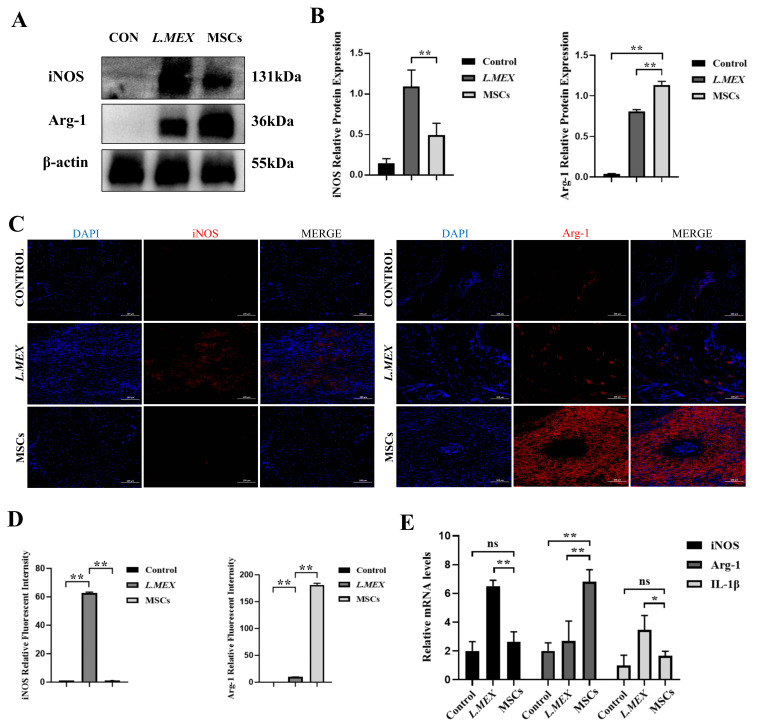
BM-MSC treatment modulates macrophage-associated polarization markers in skin lesions. (**A**) Western blots for iNOS and Arg-1. (**B**) Densitometric quantification. (**C**) Confocal immunofluorescence for iNOS (red) and Arg-1 (green); DAPI (blue). (**D**) Semi-quantitative analysis of relative fluorescence intensity for iNOS and Arg-1. (**E**) RT-qPCR for iNOS, IL-1β, and Arg-1 mRNA. Data are mean ± SD, *n* = 6. * *p* < 0.05; ** *p* < 0.01; ns, not significant. Original Western blots images can be found in the Supplementary Materials.

At the molecular level, Integrin β1 and Cytokeratin 17 protein expression in skin lesions was lower in the BM-MSC group than in untreated infected animals (*p* < 0.01), approaching control values, although mRNA changes were less pronounced (Figure 6). Tissue parasite load, measured by kDNA-targeting TaqMan qPCR, was also significantly reduced in treated mice (Figure 7). Western blot and immunofluorescence showed lower iNOS and higher Arg-1 protein in lesion tissue from treated mice (Figure 8A–D), and this pattern was reflected in qPCR data showing decreased iNOS and IL-1β alongside increased Arg-1 transcripts (Figure 8E). Therefore, systemic BM-MSC delivery reshaped the inflammatory microenvironment of established CL lesions while also reducing the local parasite burden.

## 4. Discussion

The main finding of this study was that bone marrow-derived MSCs, given intravenously to mice with established cutaneous leishmaniasis, reduced skin inflammation, improved tissue architecture, and decreased tissue parasite burden. In parallel in vitro experiments, BM-MSC co-culture shifted macrophages from an M1 toward an M2 phenotype. Together, these data supported the notion that BM-MSCs engage both the immune and reparative aspects of CL—a profile that current antiparasitic drugs lack.

The macrophage polarization shift observed in vitro fits with a broader body of work on MSC–macrophage interactions. During physiological wound healing, an initial M1 response—necessary for pathogen clearance and debris removal—gives way to M2 activation that dampens inflammation and promotes matrix remodeling [15,16]. MSCs promote skin repair by modulating macrophage function and favoring an M2-associated, pro-repair microenvironment [23,24,25]. In CL, this transition appears to stall; based on flow cytometry data, M1 dominance was maintained through at least 48 h of *L. mexicana* infection, with only partial M2 emergence at 96 h. Notably, BM-MSC co-culture increased iNOS expression in IL-4-induced M2 macrophages. Evidence indicates that macrophages expressing both M1- and M2-associated markers during inflammation resolution [26,27] and BM-MSCs can promote M2b polarization with concurrent increases in iNOS and IL-10 under M2-polarizing conditions [28]. Since multiple soluble mediators can induce M2 or M2-like macrophage polarization under different experimental settings [29,30,31,32,33], whether other stromal or epithelial-lineage cells could produce similar paracrine effects remains to be determined.

Several paracrine mediators produced by MSCs are known to promote M2 polarization. Prostaglandin E2 drives macrophages toward IL-10-producing M2-like states via cAMP [32], and MSC-derived TGF-β1 activates the Akt/FoxO1 pathway to similar effect [34]. Extracellular vesicles (EVs) from MSCs retain much of their parent cell’s immunomodulatory activity [35,36], and the specific cargo—TGF-β1, PGE2, and others—varies depending on culture conditions and the inflammatory stimulus the MSCs have received [37,38,39]. However, factors underlying the M2 shift were not identified and would require targeted conditioned-medium fractionation or mediator-specific blockade to resolve.

The reduction in skin parasite burden after BM-MSC treatment was somewhat unexpected, given that M2 macrophages are generally considered more permissive to *Leishmania* survival than M1 cells [40,41]. Perhaps intravenously administered MSCs release systemic mediators that stimulate Th1 responses in lymphoid organs, enhancing anti-parasitic immunity at sites distant from the lesion [42]. Alternatively, restoration of tissue structure may deprive the parasite of the inflammatory microenvironment it exploits. However, our study was not designed to distinguish between these mechanisms; we measured neither draining lymph node cytokine profiles nor splenic T cell subsets. Therefore, this remains an important question for follow-up work.

A somewhat puzzling observation was the discordance between protein and mRNA for Integrin β1 and Cytokeratin 17 in skin tissue; although BM-MSC treatment significantly reduced protein levels of both markers, mRNA changes were smaller and inconsistent. A similar pattern was reported in other MSC studies [25] and may reflect post-translational regulation—altered protein turnover, proteasomal degradation, or matrix-dependent stabilization—rather than transcriptional control. Whether this is a general feature of MSC-mediated tissue repair or specific to the *Leishmania* model is unclear.

The host immune background is likely to influence MSC outcomes in leishmaniasis. We used BALB/c mice, which mount an early Th2-skewed response and develop progressive, chronic infection rather than self-healing lesions [43,44,45,46]. This susceptible background may have amplified the apparent therapeutic benefit of BM-MSCs. Whether similar effects would be seen in C57BL/6 mice, which control *L. major* infection through Th1 immunity [44], or in immunocompetent models of other *Leishmania* species, remains to be tested. The question of combining BM-MSC therapy with conventional antiparasitic drugs is also open; one study reported synergy between adipose-derived MSCs and meglumine antimoniate in an *L. amazonensis* model [20], but no equivalent data exist for BM-MSCs or for *L. mexicana*.

From a translational standpoint, BM-MSC therapy is attractive precisely because it acts on the host rather than the parasite, circumventing resistance mechanisms that undermine conventional drugs. The in vivo improvements in tissue architecture and collagen remodeling observed here point to a reparative function that no current antileishmanial treatment provides [47,48,49]. Practical hurdles—scalable manufacturing, route and dosing optimization, and cost—are real, but they are not unique to this application. MSC-derived EVs may eventually offer a cell-free alternative that is easier to standardize and store [35].

This study had several limitations. Mechanistic evidence for M2 reprogramming was based on indirect co-culture data; identifying the responsible paracrine mediators will require more targeted approaches. Parasite burden was assessed at a single endpoint, so we cannot say whether the reduction is sustained. Systemic immune parameters—serum cytokines, T cell subsets, and splenic responses—were not measured, leaving the broader immunological picture incomplete. Finally, the study used a single BM-MSC source and dosing regimen; both variables may affect outcomes substantially and should be explored systematically.

## 5. Conclusions

Bone marrow-derived MSCs reduced inflammation, improved skin histology, and decreased tissue parasite burden in a murine model of cutaneous leishmaniasis, while also modulating macrophage polarization in M1- and M2-stimulated macrophage cultures. These effects address aspects of CL pathology—persistent non-healing lesions, fibrotic remodeling, and a dysregulated immune microenvironment—that standard chemotherapy does not target. The findings provide a rationale for exploring BM-MSC therapy as an adjunct to conventional treatment and point to the need for mechanistic studies identifying the paracrine factors responsible for macrophage reprogramming, as well as combination and longitudinal experiments to establish durability and clinical feasibility.

## Figures and Tables

**Table 1:** Primer sequences used in RT-qPCR and parasite burden assays.

Gene	Primer Sequence (5′–3′)
β-actin	F: TGGAATCCTGTGGCATCCATGAAAC R: TAAAACGCAGCTCAGTAACAGTCCG
Cytokeratin 17	F: ACCATCCGCCAGTTTACCTC R: CTACCCAGGCCACTAGCTGA
Integrin β1	F: CGTGGTTGCCGGAATTGTTC R: ACCAGCTTTACGTCCATAGTTTG
iNOS	F: CAGCTGGGCTGTACAAACCTT R: CATTGGAAGTGAAGCGTTTCG
Arg-1	F: AACACTCCCCTGACAACCA R: CATCACCTTGCCAATCCC

## Data Availability

The original contributions presented in this study are included in the article/Supplementary Material. Further inquiries can be directed to the corresponding authors. The data are not publicly available due to ongoing studies.

## Supplementary Materials

The following supporting information can be downloaded at: https://www.mdpi.com/article/10.3390/biom16060897/s1, File S1: Original Western blot images.

## Abbreviations

The following abbreviations are used in this manuscript:

MSCs	Mesenchymal stem cells
CL	Cutaneous leishmaniasis
BM-MSCs	Bone marrow-derived MSCs
EVs	Extracellular vesicles
iNOS	Inducible nitric oxide synthase
Arg-1	Arginase-1
DAPI	4′,6-diamidino-2-phenylindole

## References

[1] De Vries H.J.C., Schallig H.D. (2022). Cutaneous Leishmaniasis: A 2022 Updated Narrative Review into Diagnosis and Management Developments. Am. J. Clin. Dermatol..

[2] Aronson N.E., Musa A.M., Satoskar A.R. (2026). Leishmaniasis. N. Engl. J. Med..

[3] Oryan A., Akbari M. (2016). Worldwide risk factors in leishmaniasis. Asian Pac. J. Trop. Med..

[4] Liu G., Wu Y., Wang L., Liu Y., Huang W., Li Y., Gao M., Kastelic J., Barkema H.W., Xia Z. (2023). Re-emergence of canine *Leishmania infantum* infection in mountain areas of Beijing. One Health Adv..

[5] Khanra S., Das S., Sarraf N.R., Datta S., Das A.K., Manna M., Roy S. (2022). Antimony resistance mechanism in genetically different clinical isolates of Indian Kala-azar patients. Front. Cell Infect. Microbiol..

[6] Frézard F., Aguiar M.M.G., Ferreira L.A.M., Ramos G.S., Santos T.T., Borges G.S.M., Vallejos V.M.R., De Morais H.L.O. (2022). Liposomal Amphotericin B for Treatment of Leishmaniasis: From the Identification of Critical Physicochemical Attributes to the Design of Effective Topical and Oral Formulations. Pharmaceutics.

[7] Siddiqui N.A., Ansari M.Z., Sinha S.K., Pal B., Singh A.K., Singh S.K., Topno R.K., Rabi Das V.N., Pandey K. (2024). Treatment Outcomes of Single-Dose Liposomal Amphotericin B-Treated Visceral Leishmaniasis Patients and Factors Affecting Outcome in Bihar, India. Am. J. Trop. Med. Hyg..

[8] Astman N., Arbel C., Katz O., Barzilai A., Solomon M., Schwartz E. (2024). Tolerability and Safety of Miltefosine for the Treatment of Cutaneous Leishmaniasis. Trop. Med. Infect. Dis..

[9] Sundar S., Chakravarty J. (2015). An update on pharmacotherapy for leishmaniasis. Expert. Opin. Pharmacother..

[10] Sadeghian G., Nilfroushzadeh M.A., Iraji F. (2007). Efficacy of local heat therapy by radiofrequency in the treatment of cutaneous leishmaniasis, compared with intralesional injection of meglumine antimoniate. Clin. Exp. Dermatol..

[11] Aronson N.E., Wortmann G.W., Byrne W.R., Howard R.S., Bernstein W.B., Marovich M.A., Polhemus M.E., Oster C.N., Spooner A., Williams C. (2010). A randomized controlled trial of local heat therapy versus intravenous sodium stibogluconate for the treatment of cutaneous *Leishmania major* infection. PLoS Negl. Trop. Dis..

[12] Shamsi Meymandi S., Zandi S., Aghaie H., Hooshmand B. (2011). Efficacy of CO_2_ laser for treatment of anthroponotic cutaneous leishmaniasis, compared with combination of cryotherapy and intralesional meglumine antimoniate. J. Eur. Acad. Dermatol. Venereol..

[13] Mosleh I.M., Geith E., Natsheh L., Abdul-Dayem M., Abotteen N. (2008). Efficacy of a weekly cryotherapy regimen to treat *Leishmania major* cutaneous leishmaniasis. J. Am. Acad. Dermatol..

[14] Naji A., Eitoku M., Favier B., Deschaseaux F., Rouas-Freiss N., Suganuma N. (2019). Biological functions of mesenchymal stem cells and clinical implications. Cell Mol. Life Sci..

[15] Liu Y., Zou X., Chai Y., Yao Y. (2014). Macrophage polarization in inflammatory diseases. Int. J. Biol. Sci..

[16] Sharifiaghdam M., Shaabani E., Faridi-Majidi R., De Smedt S.C., Braeckmans K., Fraire J.C. (2022). Macrophages as a therapeutic target to promote diabetic wound healing. Mol. Ther..

[17] Dameshghi S., Zavaran-Hosseini A., Soudi S., Shirazi F.J., Nojehdehi S., Hashemi S.M. (2016). Mesenchymal stem cells alter macrophage immune responses to *Leishmania major* infection in both susceptible and resistance mice. Immunol. Lett..

[18] Khosrowpour Z., Hashemi S.M., Mohammadi-Yeganeh S., Zavaran Hosseini A., Sharif Paghaleh N., Nojehdehi S. (2017). Pretreatment of mesenchymal stem cells with *Leishmania major* soluble antigens induces anti-inflammatory properties in mouse peritoneal macrophages. J. Cell. Biochem..

[19] Navard S.H., Rezvan H., Haddad M.H.F., Khosravi A. (2020). Therapeutic effects of mesenchymal stem cells on cutaneous leishmaniasis lesions caused by *Leishmania major*. J. Glob. Antimicrob. Resist..

[20] Zanganeh E., Soudi S., Zavaran Hosseini A., Hashemi S.M. (2019). Repeated intravenous injection of adipose tissue-derived mesenchymal stem cells enhances Th1 immune responses in *Leishmania major*-infected BALB/c mice. Immunol. Lett..

[21] Pereira J.C., Ramos T.D., Silva J.D., Capichoni S., Souza S.A.L., Takyia C.M., Madi K., Rezende-Castro P.A., Dos Santos C.C., Rocco P.R.M. (2017). Effects of bone marrow mesenchymal stromal cell therapy in experimental cutaneous leishmaniasis in BALB/c mice induced by *Leishmania amazonensis*. Front. Immunol..

[22] Ramos T.D., Silva J.D., Da Fonseca-Martins A.M., Pratti J.E.S., Firmino-Cruz L., Pereira J.C., Sousa-Batista A.J., Gutfilen B., De Souza S.A.L., Rocco P.R.M. (2020). Combined therapy with adipose tissue-derived mesenchymal stromal cells and meglumine antimoniate controls lesion development and parasite load in murine cutaneous leishmaniasis caused by *Leishmania amazonensis*. Stem Cell Res. Ther..

[23] Italiani P., Mazza E.M.C., Lucchesi D., Cifola I., Gemelli C., Grande A., Battaglia C., Bicciato S., Boraschi D. (2014). Transcriptomic profiling of the development of the inflammatory response in human monocytes *in vitro*. PLoS ONE.

[24] Liu Y., Zhang M., Liao Y., Chen H., Su D., Tao Y., Li J., Luo K., Wu L., Zhang X. (2023). Human umbilical cord mesenchymal stem cell-derived exosomes promote murine skin wound healing by neutrophil and macrophage modulations revealed by single-cell RNA sequencing. Front. Immunol..

[25] Zhang Q., Su W.R., Shi S.H., Witek L., Yao Q.Q., Wang J.L., Deng C. (2010). Human gingiva-derived mesenchymal stem cells elicit polarization of M2 macrophages and enhance cutaneous wound healing. Stem Cells.

[26] Bystrom J., Evans I., Newson J., Stables M., Toor I., Van Rooijen N., Crawford M., Colville-Nash P., Dalli J., Gilroy D.W. (2008). Resolution-phase macrophages possess a unique inflammatory phenotype that is controlled by cAMP. Blood.

[27] Routley C.E., Ashcroft G.S. (2009). Effect of estrogen and progesterone on macrophage activation during wound healing. Wound Repair Regen..

[28] Philipp D., Suhr L., Wahlers T., Choi Y.H., Paunel-Görgülü A. (2018). Preconditioning of bone marrow-derived mesenchymal stem cells highly strengthens their potential to promote IL-6-dependent M2b polarization. Stem Cell Res. Ther..

[29] Chen S., Saeed A.F.U.H., Liu Q., Jiang Q., Xu H., Xiao G.G., Rao L., Duo Y. (2023). Macrophages in immunoregulation and therapeutics. Signal Transduct. Target. Ther..

[30] Chen G.H., Olszewski M.A., McDonald R.A., Wells J.C., Paine R., Huffnagle G.B., Toews G.B. (2007). Role of granulocyte macrophage colony-stimulating factor in host defense against pulmonary *Cryptococcus neoformans* infection during murine allergic bronchopulmonary mycosis. Am. J. Pathol..

[31] Kuroda E., Ho V., Ruschmann J., Antignano F., Hamilton M., Rauh M.J., Antov A., Flavell R.A., Sly L.M., Krystal G. (2009). SHIP represses the generation of IL-3-induced M2 macrophages by inhibiting IL-4 production from basophils. J. Immunol..

[32] MacKenzie K.F., Clark K., Naqvi S., McGuire V.A., Nöehren G., Kristariyanto Y., van den Bosch M., Mudaliar M., McCarthy P.C., Pattison M.J. (2013). PGE2 induces macrophage IL-10 production and a regulatory-like phenotype via a protein kinase A–SIK–CRTC3 pathway. J. Immunol..

[33] Roca H., Varsos Z.S., Sud S., Craig M.J., Ying C., Bhatt S., Pienta K.J. (2009). CCL2 and interleukin-6 promote survival of human CD11b+ peripheral blood mononuclear cells and induce M2-type macrophage polarization. J. Biol. Chem..

[34] Liu F., Qiu H., Xue M., Zhang S., Zhang X., Xu J., Chen J., Yang Y., Xie J. (2019). MSC-secreted TGF-β regulates lipopolysaccharide-stimulated macrophage M2-like polarization via the Akt/FoxO1 pathway. Stem Cell Res. Ther..

[35] He X., Dong Z., Cao Y., Wang H., Liu S., Liao L., Jin Y., Yuan L., Li B. (2019). MSC-Derived Exosome Promotes M2 Polarization and Enhances Cutaneous Wound Healing. Stem Cells Int..

[36] Zhou Y., Yamamoto Y., Xiao Z., Ochiya T. (2019). The immunomodulatory functions of mesenchymal stromal/stem cells mediated via paracrine activity. J. Clin. Med..

[37] Li N., Hua J. (2017). Interactions between mesenchymal stem cells and the immune system. Cell Mol. Life Sci..

[38] Mardpour S., Hamidieh A.A., Taleahmad S., Sharifzad F., Taghikhani A., Baharvand H. (2019). Interaction between mesenchymal stromal cell-derived extracellular vesicles and immune cells by distinct protein content. J. Cell. Physiol..

[39] Madrigal M., Rao K.S., Riordan N.H. (2014). A review of therapeutic effects of mesenchymal stem cell secretions and induction of secretory modification by different culture methods. J. Transl. Med..

[40] Rossi M., Fasel N. (2018). How to master the host immune system? *Leishmania* parasites have the solutions!. Int. Immunol..

[41] Dayakar A., Chandrasekaran S., Kuchipudi S.V., Kalangi S.K. (2019). Cytokines: Key determinants of resistance or disease progression in visceral leishmaniasis: Opportunities for novel diagnostics and immunotherapy. Front. Immunol..

[42] Weiss A.R.R., Dahlke M.H. (2019). Immunomodulation by mesenchymal stem cells (MSCs): Mechanisms of action of living, apoptotic, and dead MSCs. Front. Immunol..

[43] Paton H., Sarkar P., Gurung P. (2025). An overview of host immune responses against *Leishmania* spp. infections. Hum. Mol. Genet..

[44] Scott P., Novais F.O. (2016). Cutaneous leishmaniasis: Immune responses in protection and pathogenesis. Nat. Rev. Immunol..

[45] Poudel B., Yorek M.S., Mazgaeen L., Brown S.A., Kanneganti T.D., Gurung P. (2020). Acute IL-4 Governs Pathogenic T Cell Responses during *Leishmania major* Infection. ImmunoHorizons.

[46] Tiwari R., Kumar A., Singh V.K., Rajneesh, Chauhan S.B., Sundar S., Nylén S., Engwerda C., Kumar R. (2024). The development and maintenance of immunity against visceral leishmaniasis. Front. Immunol..

[47] Miranda M.M., Panis C., da Silva S.S., Tatakihara V.L.H., Maçula A.J., Viegas S.L., Pinge-Filho P., Yamada-Ogatta S.F., Yamauchi L.M. (2015). Kaurenoic acid possesses leishmanicidal activity by triggering a NLRP12/IL-1β/cNOS/NO pathway. Mediat. Inflamm..

[48] Sahebi K., Shahsavani F., Mehravar F., Nozad-Charoudeh H., Hojati V., Darijani M.M., Mirzaei M. (2024). *In vitro* and *in vivo* anti-parasitic activity of curcumin nanoemulsion on *Leishmania major* (MRHO/IR/75/ER). BMC Complement. Med. Ther..

[49] Bhattacharjee A., Bagchi A., Sarkar S., Bhattacharya A., Lahiri D., Roy M., Bhattacharya S. (2024). Repurposing approved protein kinase inhibitors as potent anti-leishmanials targeting *Leishmania* MAP kinases. Life Sci..

