# Supplementary material for: Bone Marrow-Derived Mesenchymal Stem Cells Alleviate Cutaneous Leishmaniasis by Promoting M2 Macrophage Polarization and Skin Tissue Repair in a Murine Model
Source: Biomolecules. 2026 Jun 17;16(6):897. doi: 10.3390/biom16060897 (PMC13297385; doi:10.3390/biom16060897)
Supplement: Supplementary file 1 [file biomolecules-16-00897-s001.zip › biomolecules-4324994-supplementary.pdf]

Figure2-B

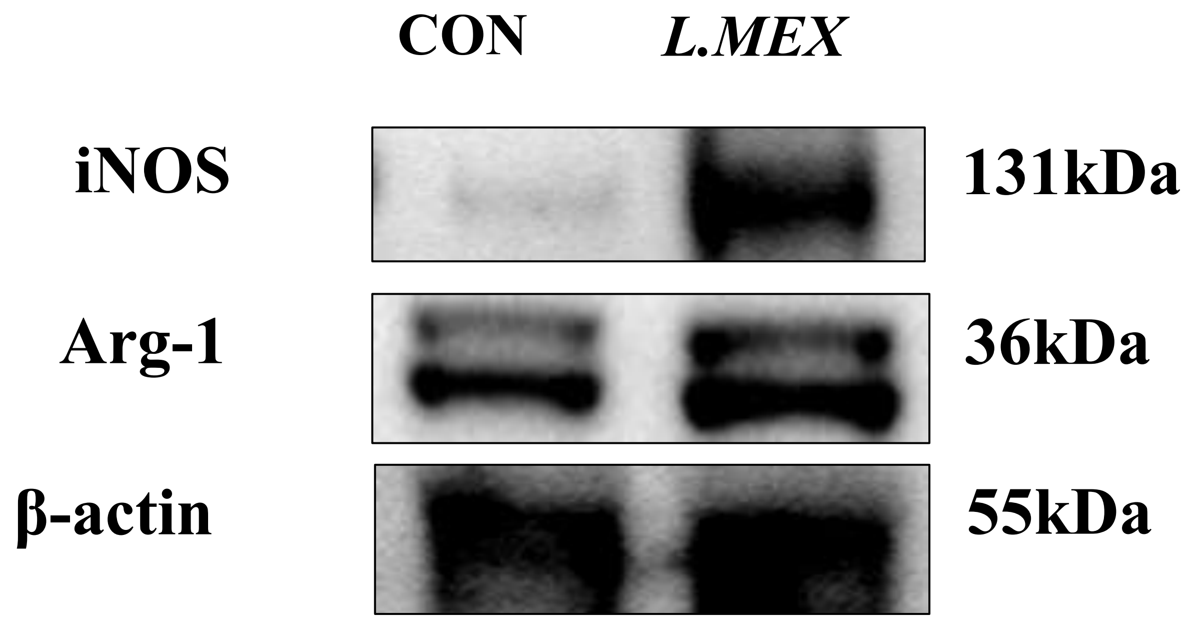

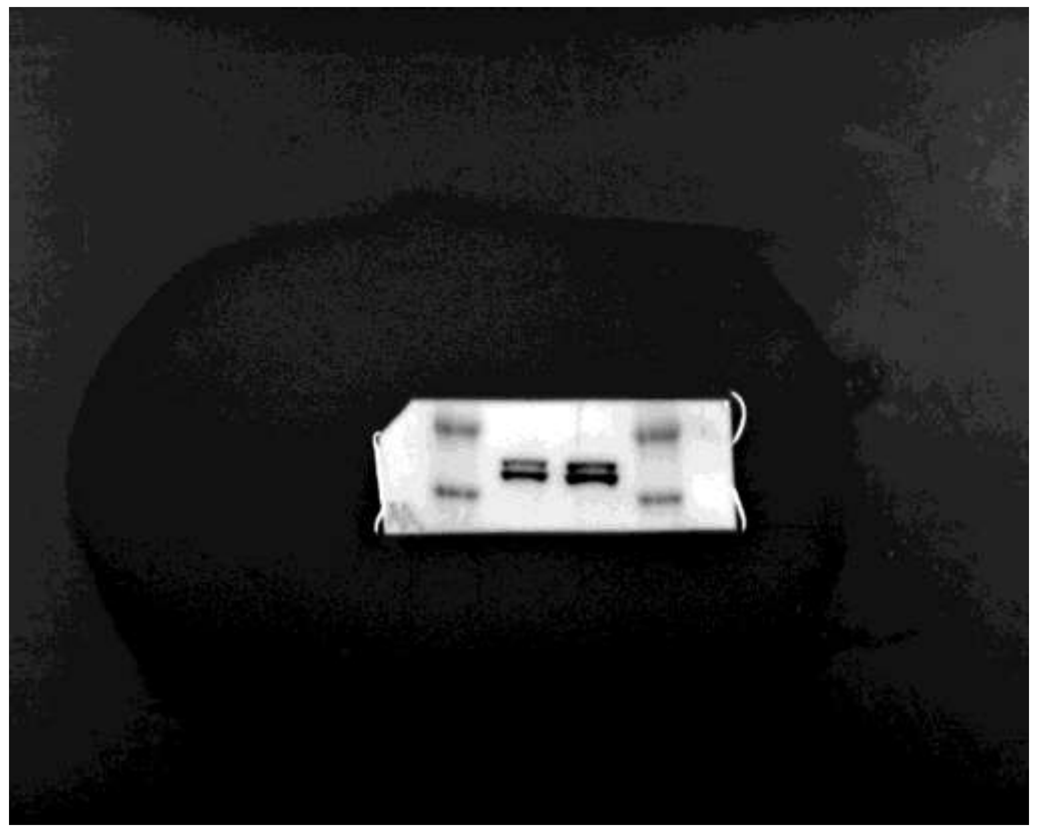

Arg-1

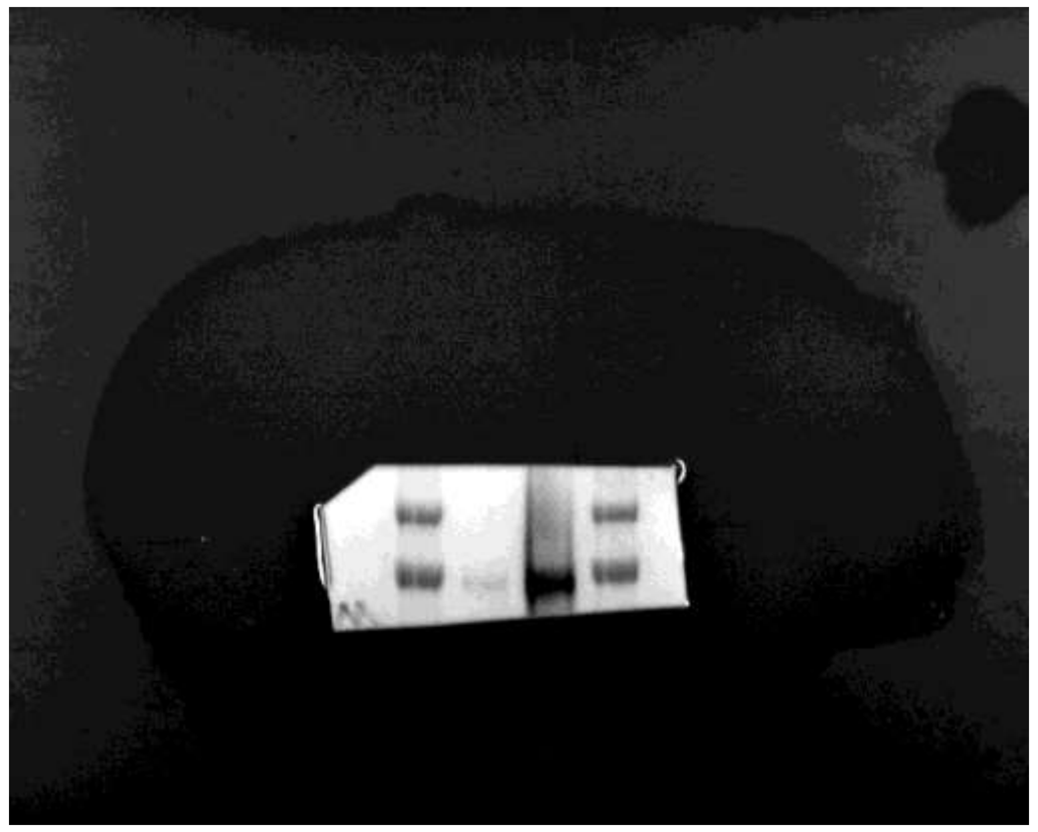

iNOS

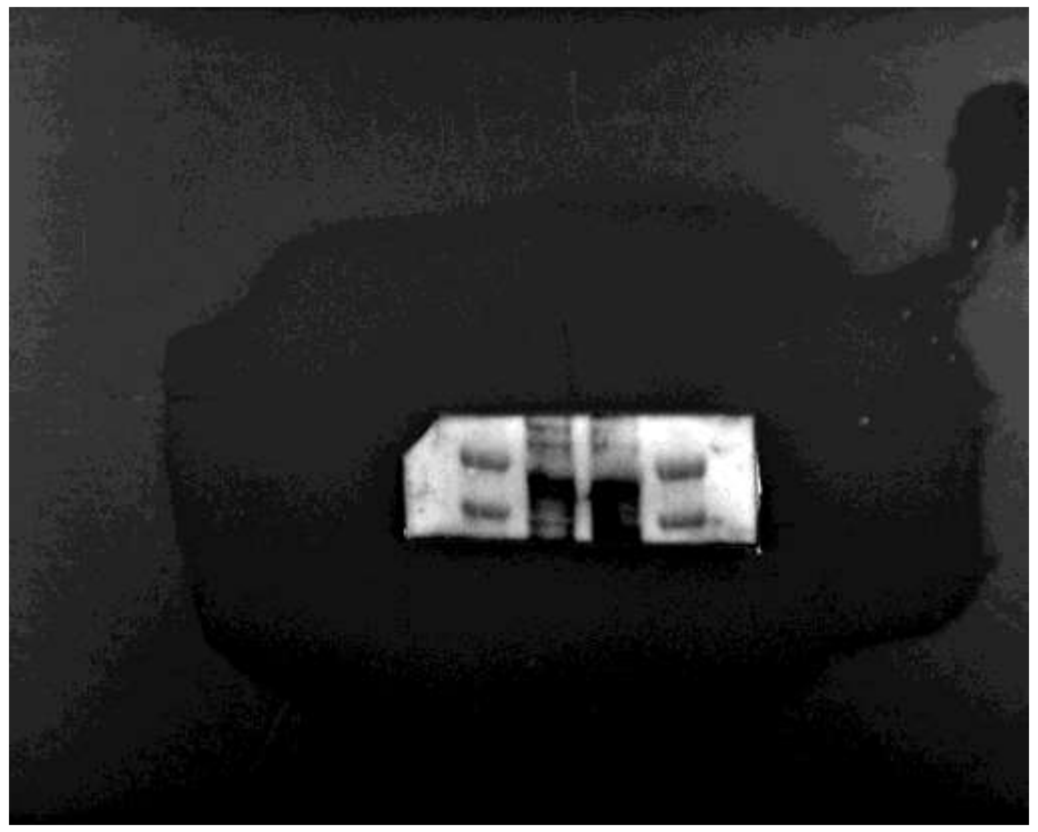

β-actin

Figure3-B

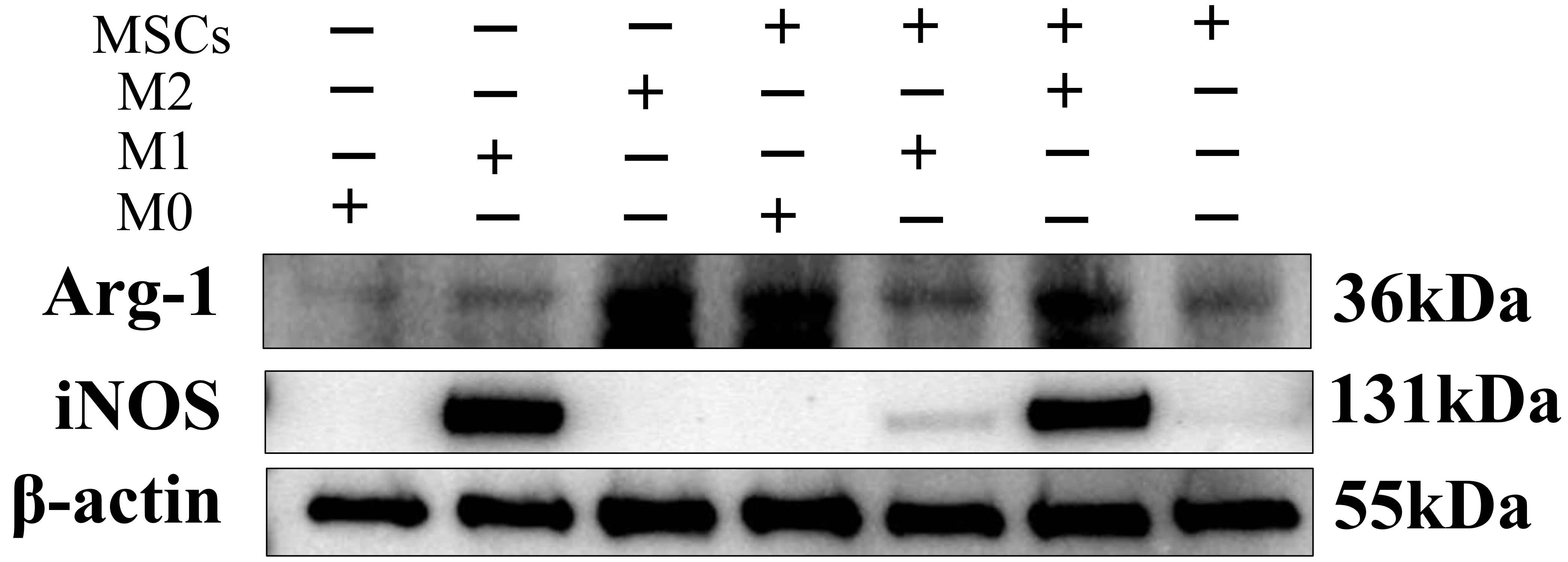

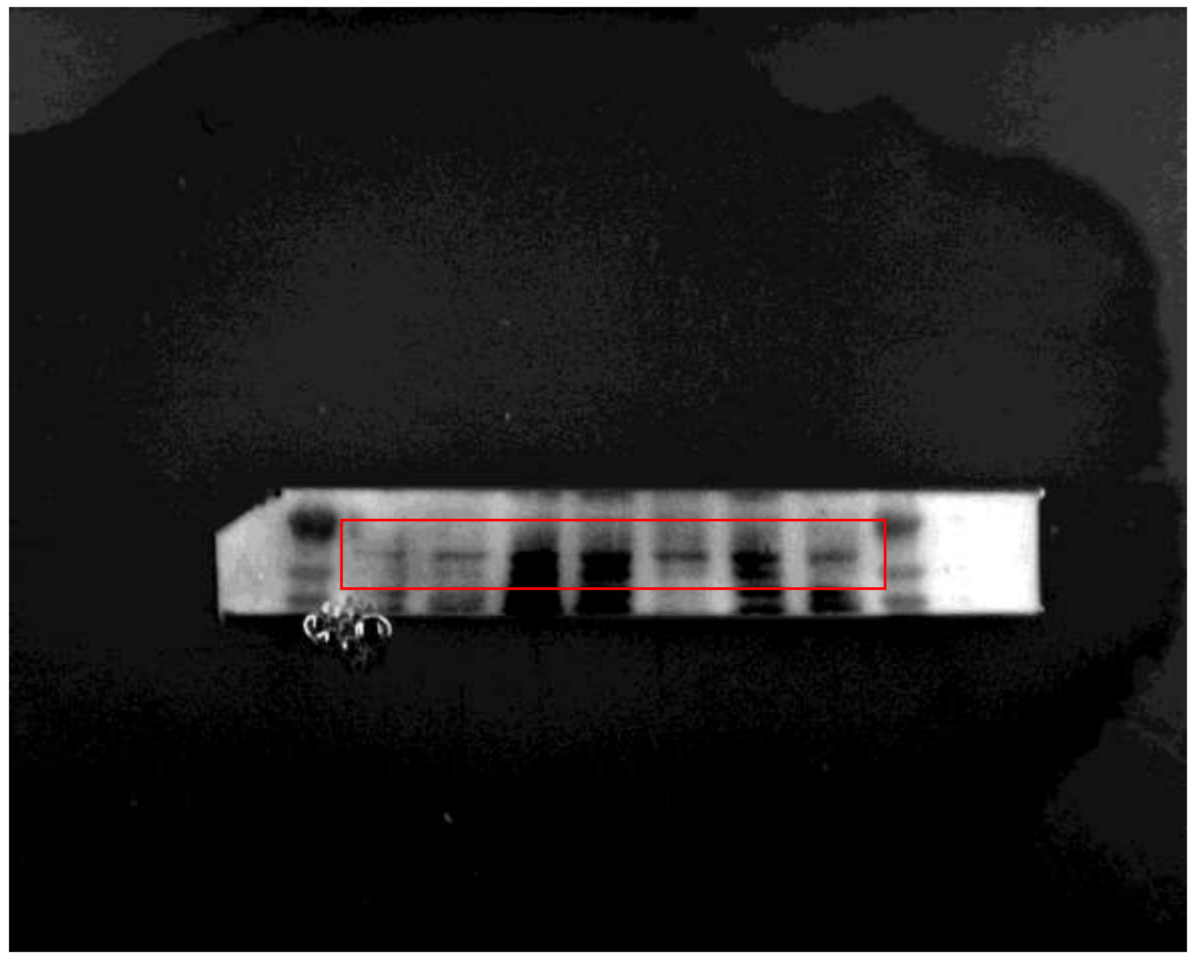

Arg-1

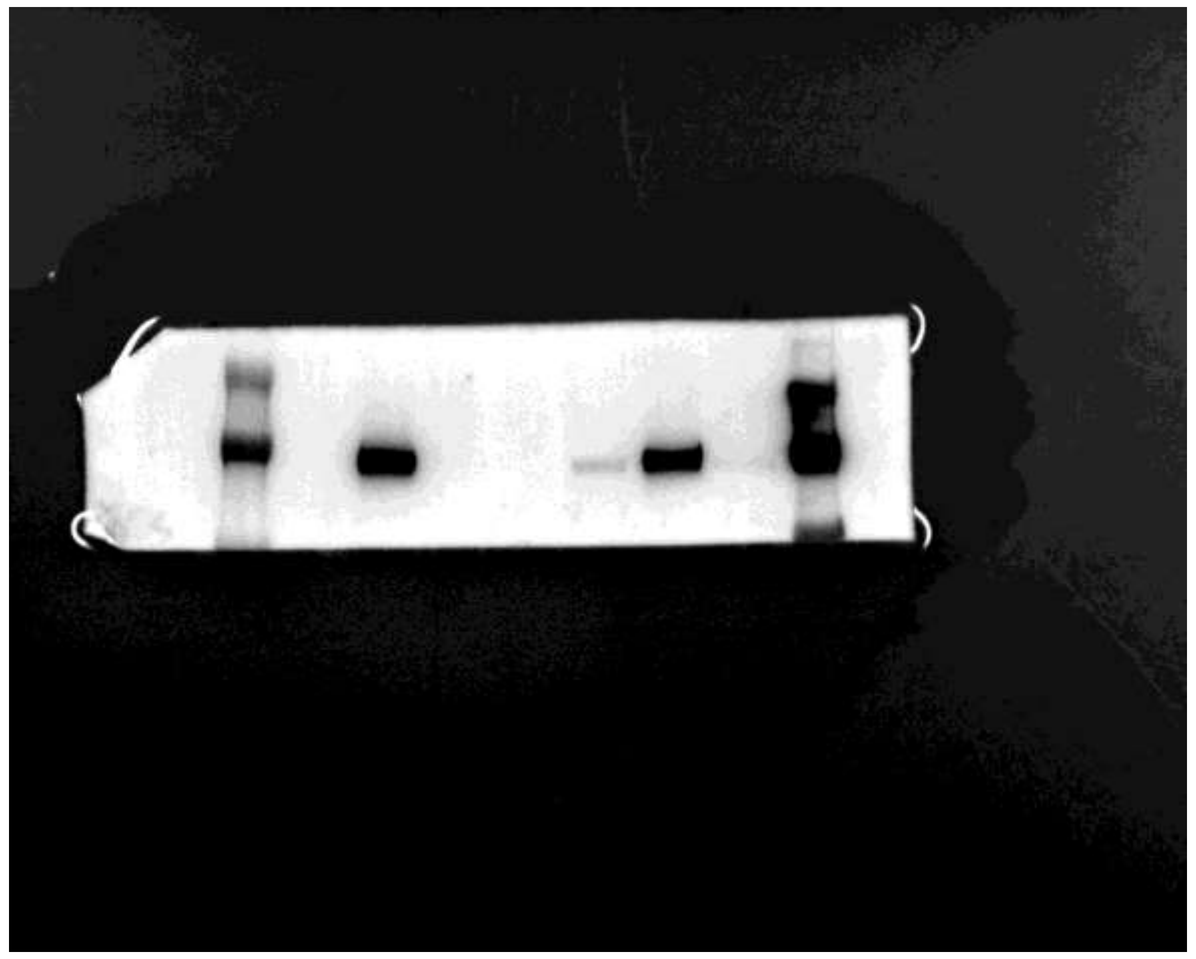

iNOS

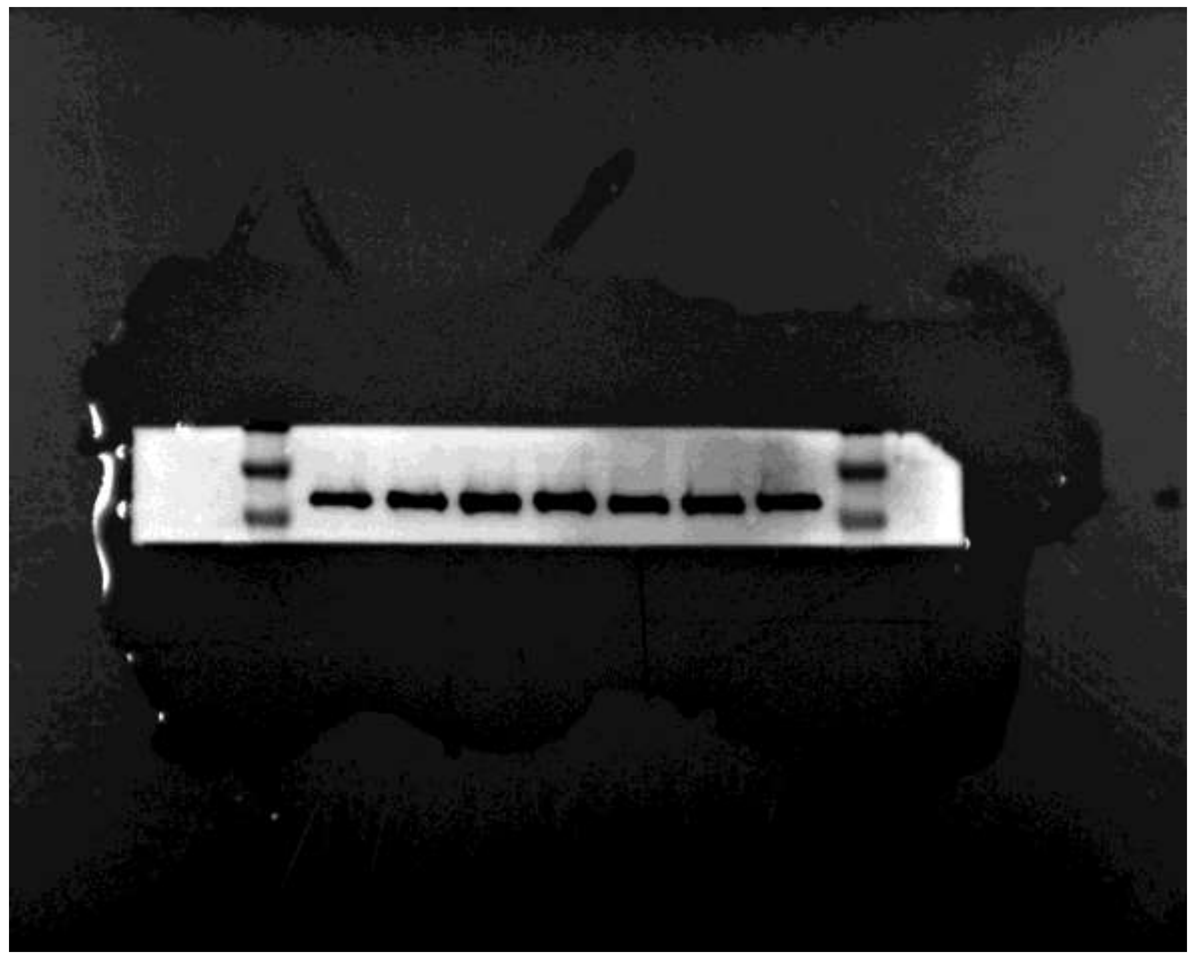

β-actin

Figure4-B

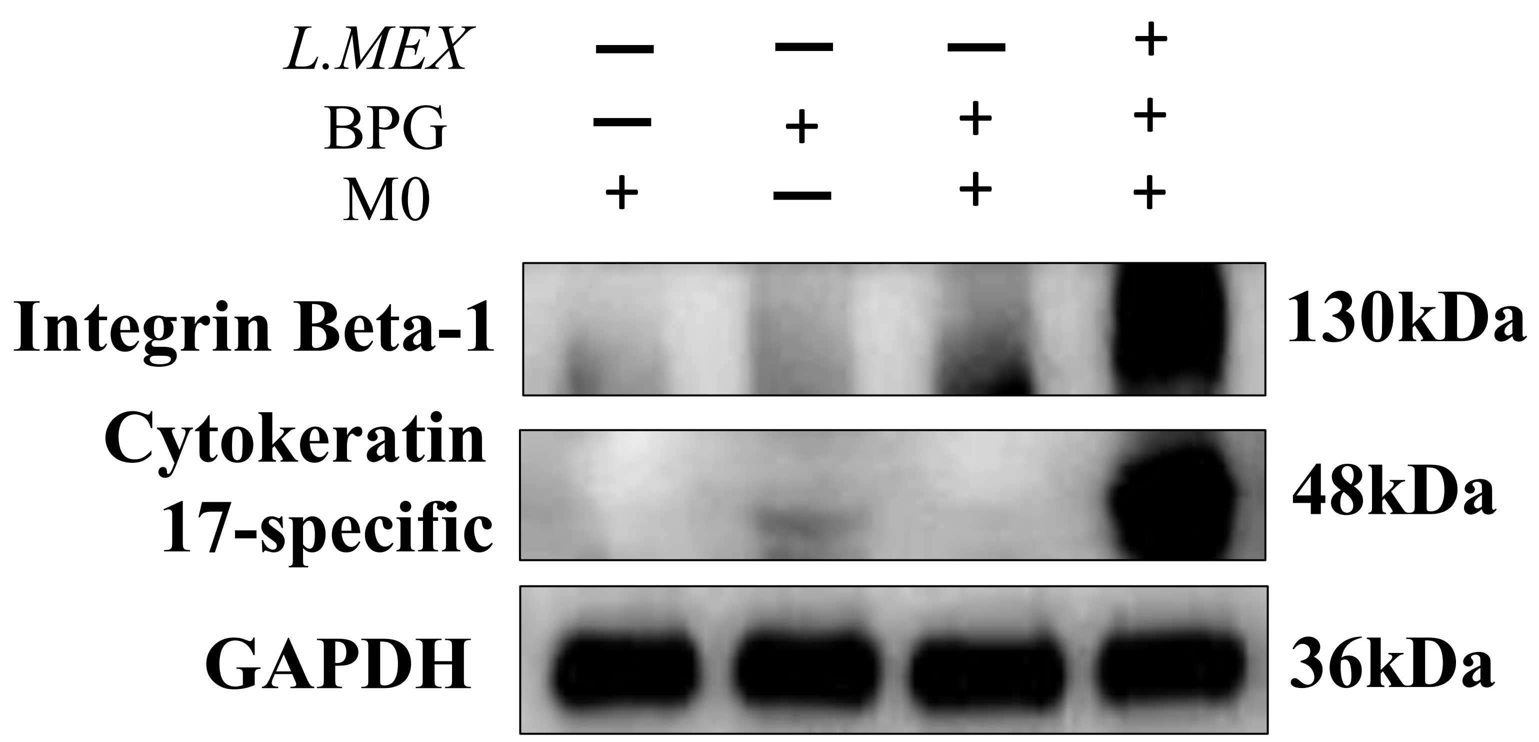

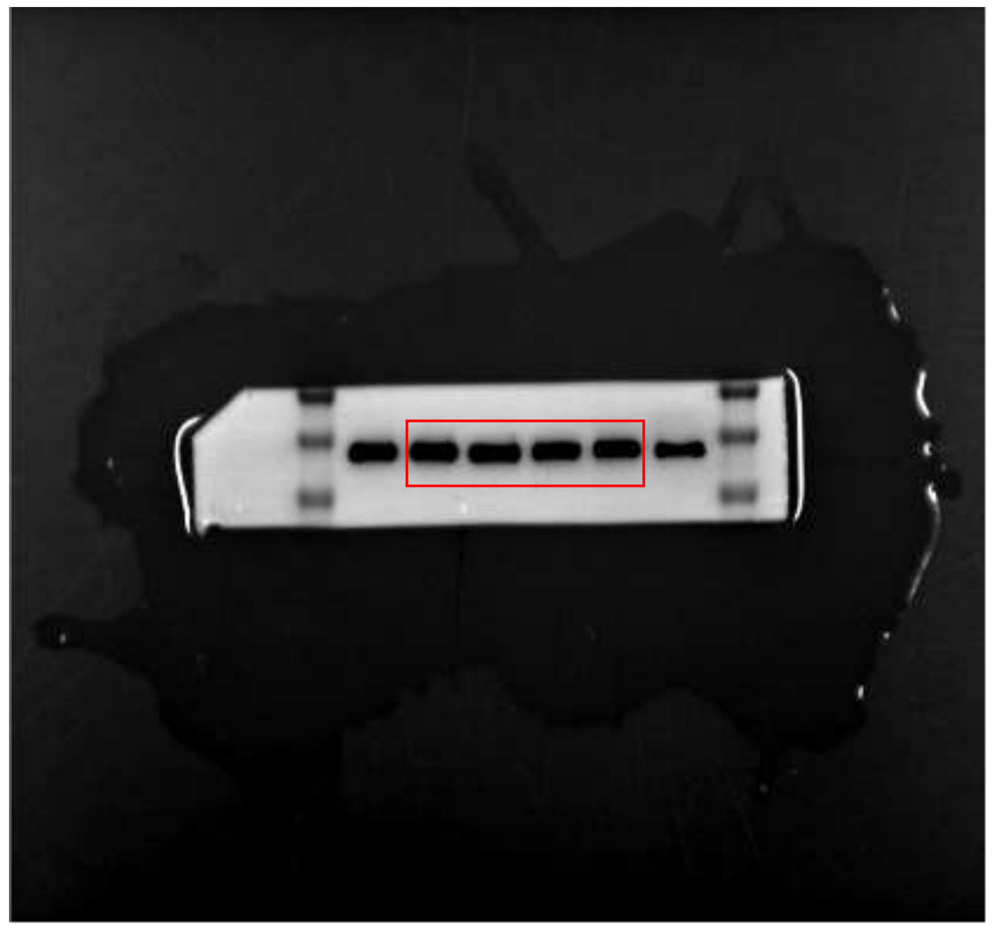

GAPDH

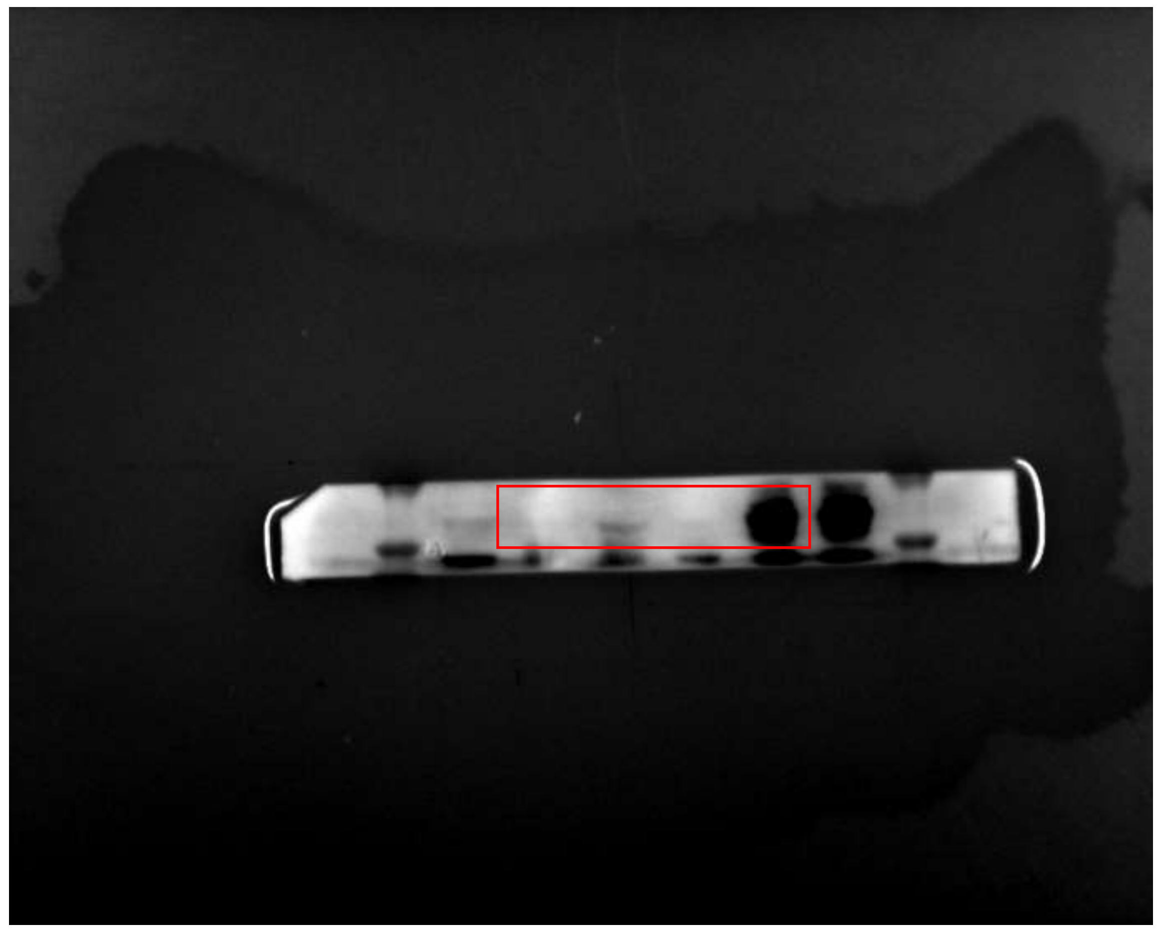

Cytokeratin
17-specific

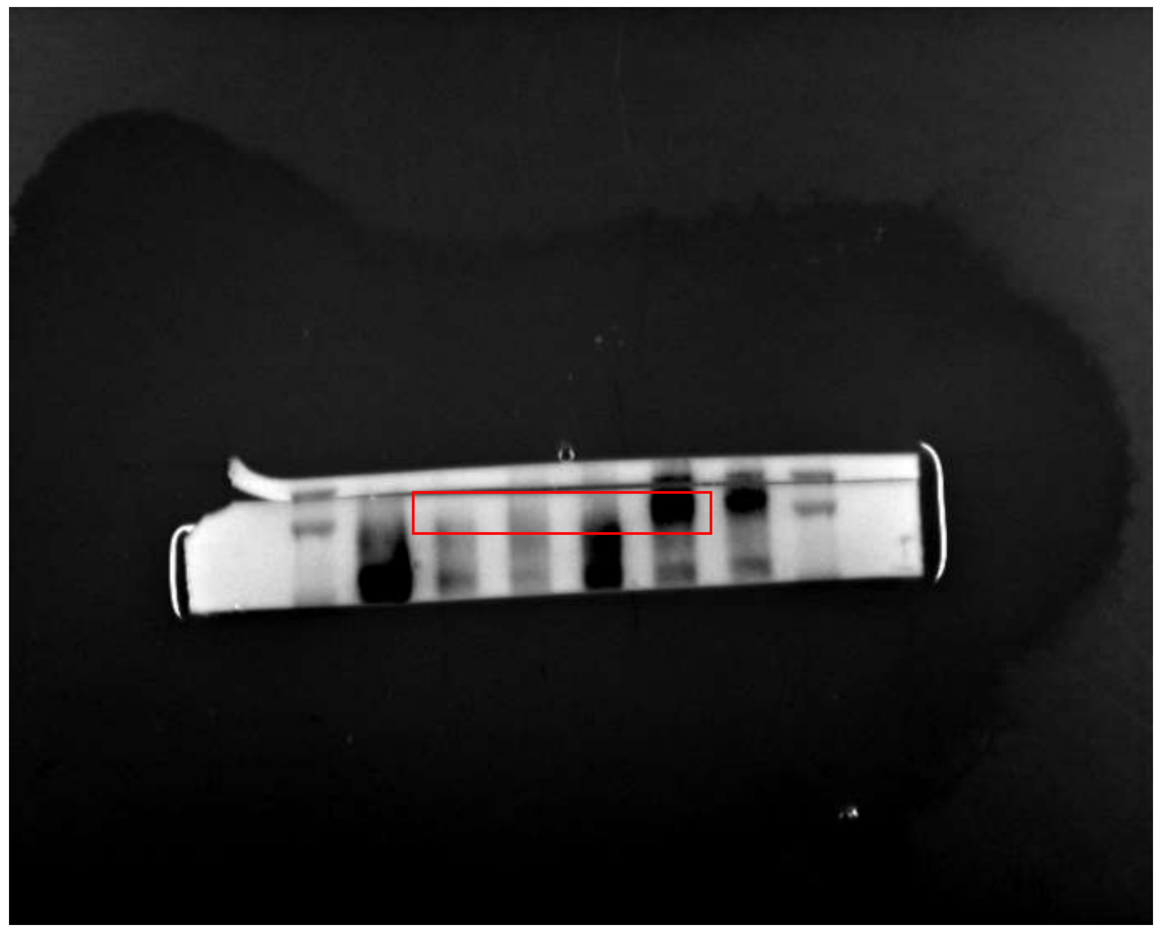

Integrin Beta-1

Figure6-B

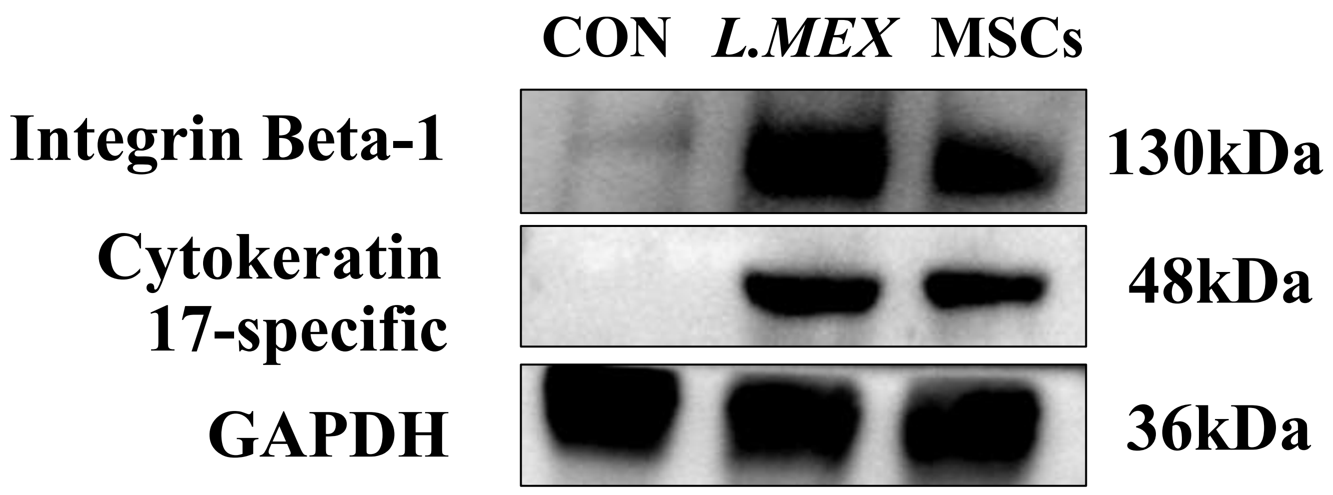

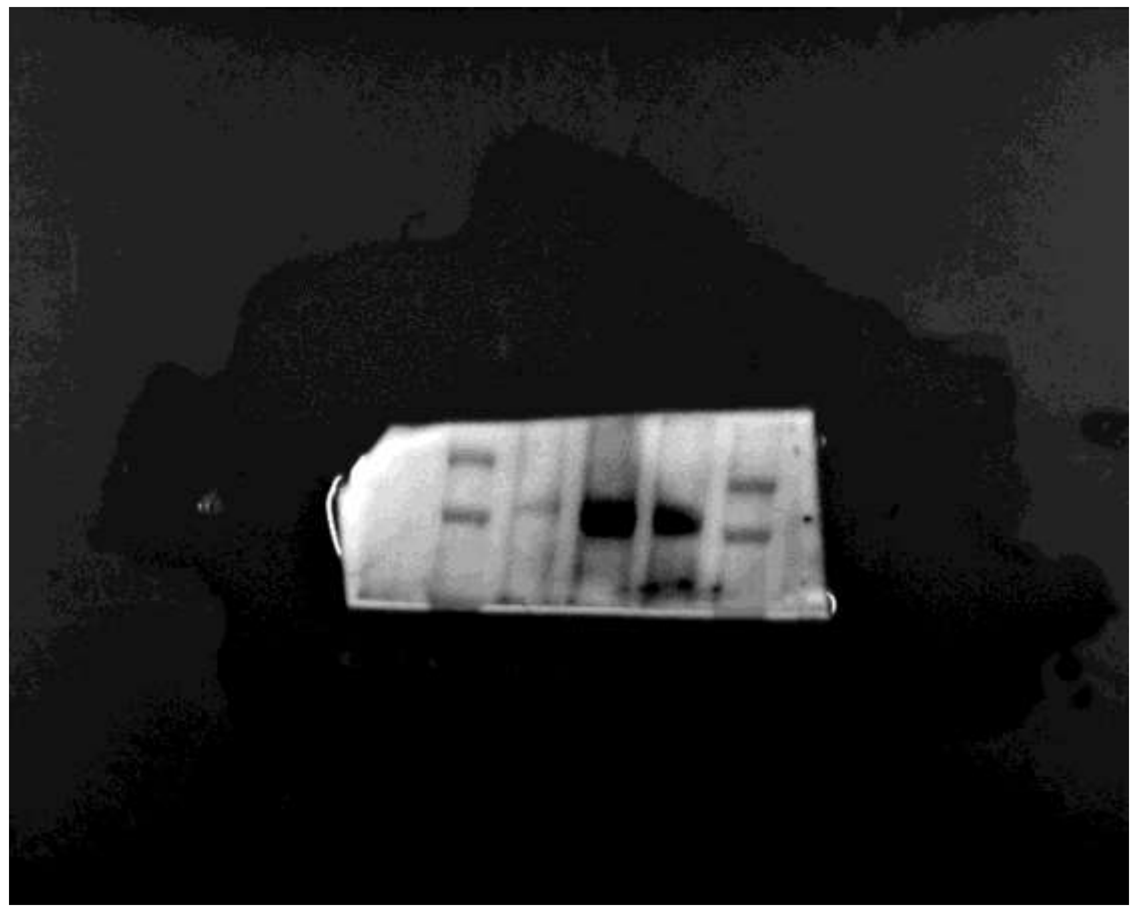

Integrin Beta-1

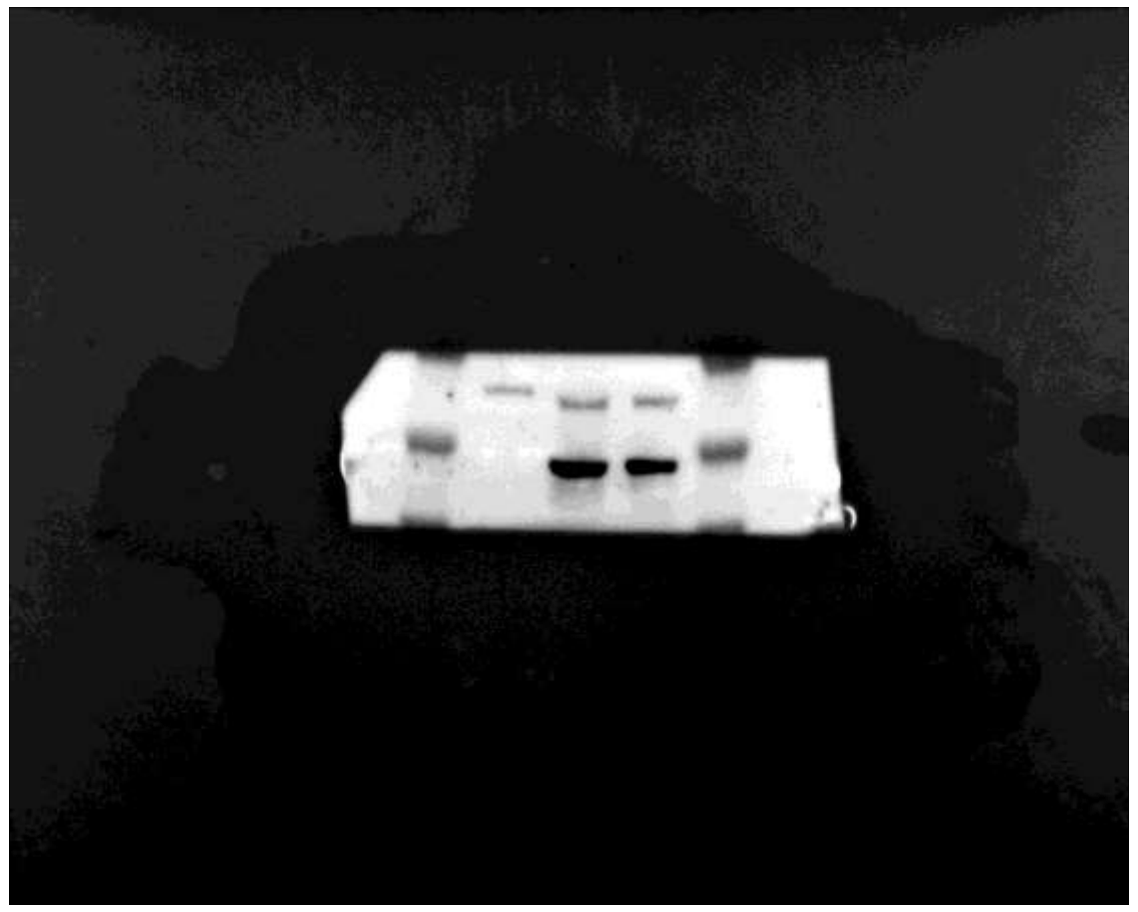

Cytokeratin 17-specific

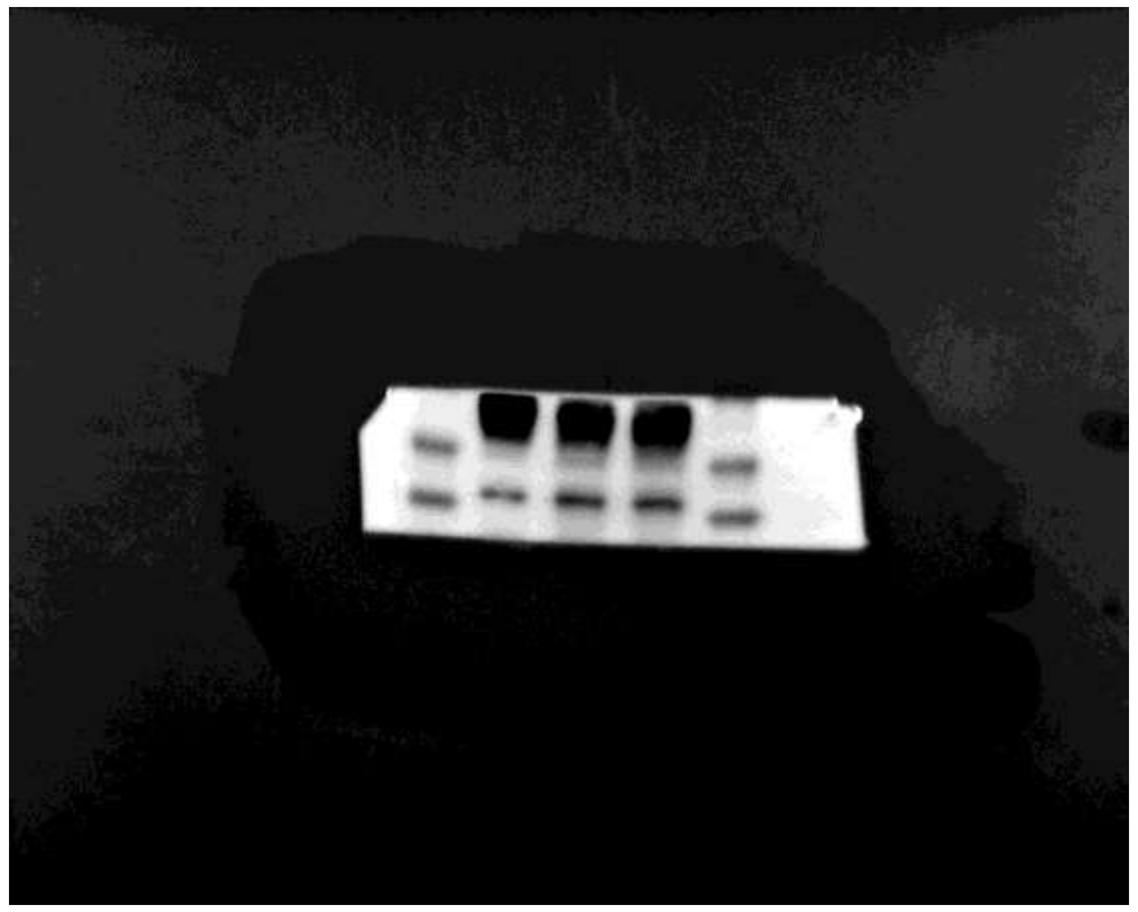

GAPDH

Figure8-A

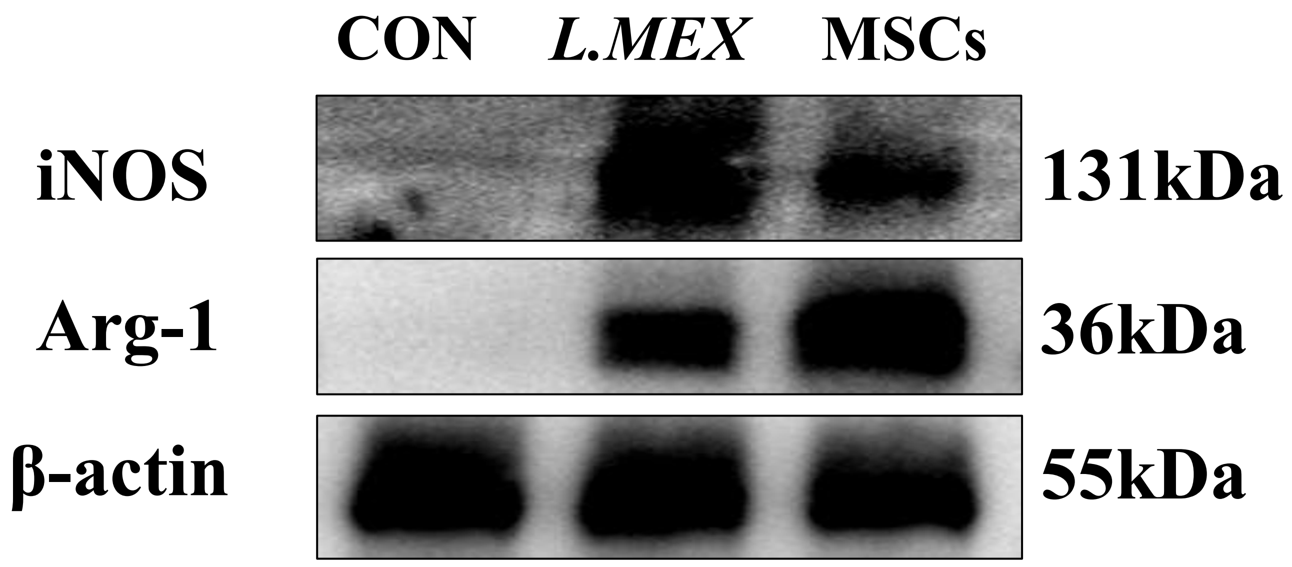

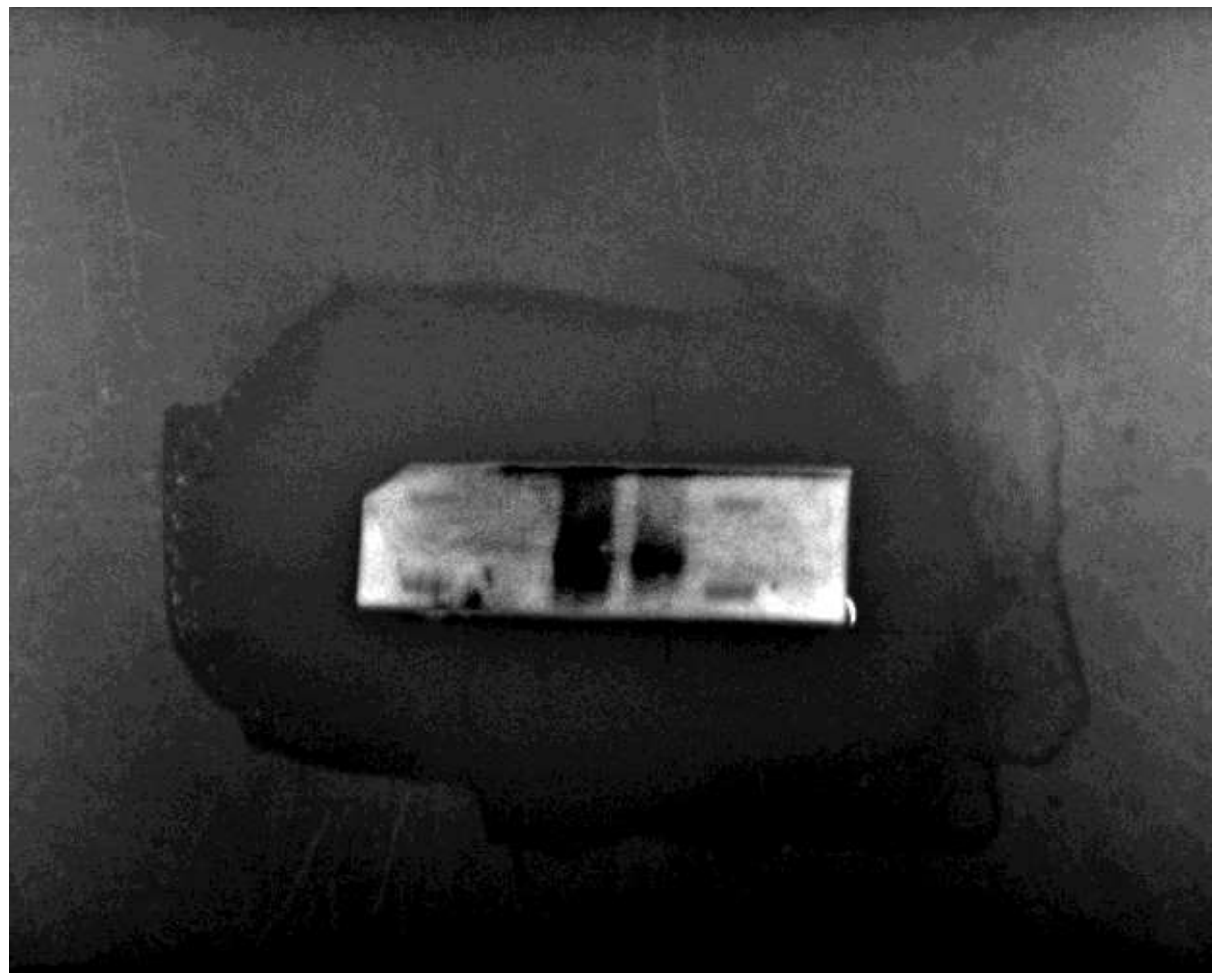

iNOS

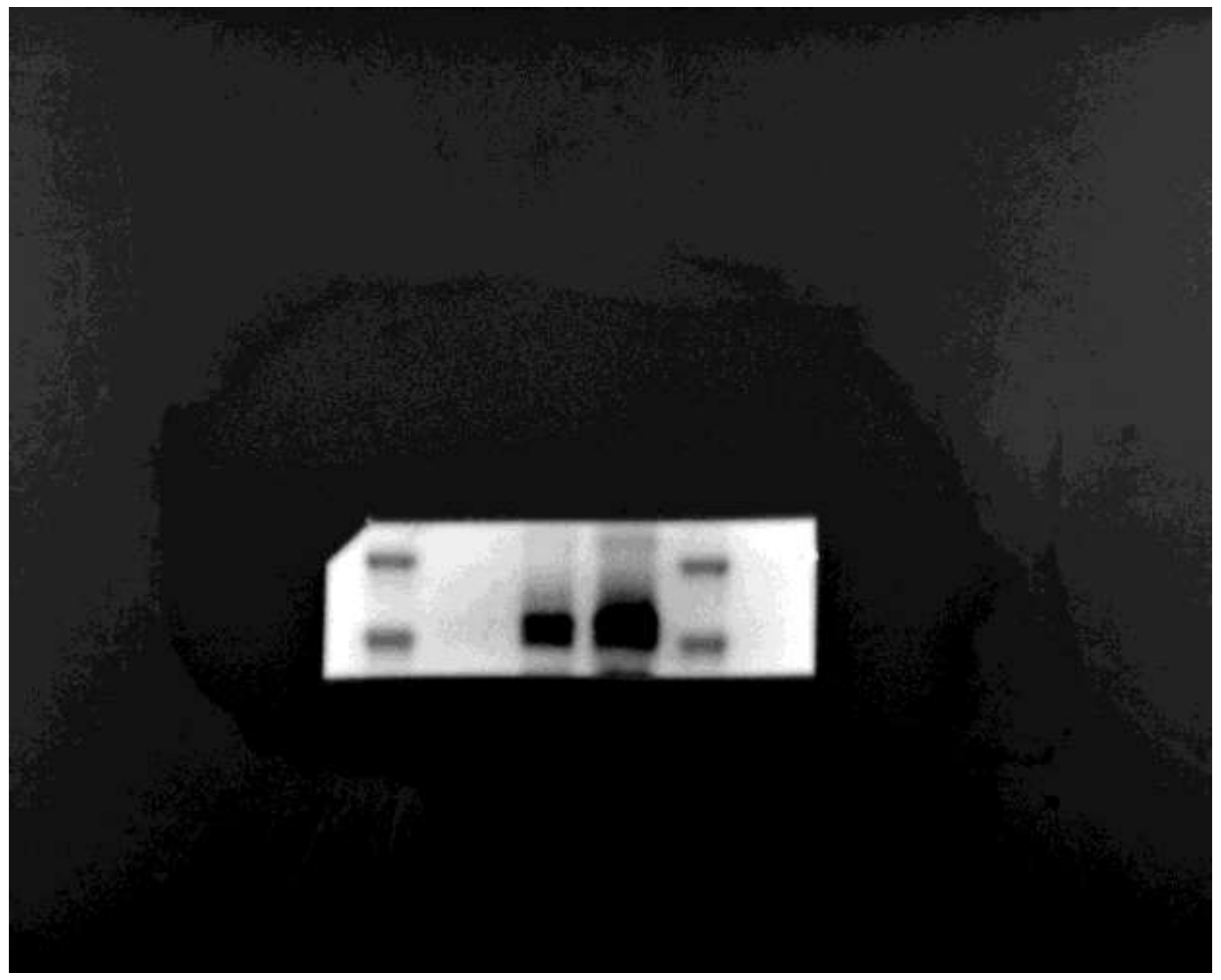

Arg-1

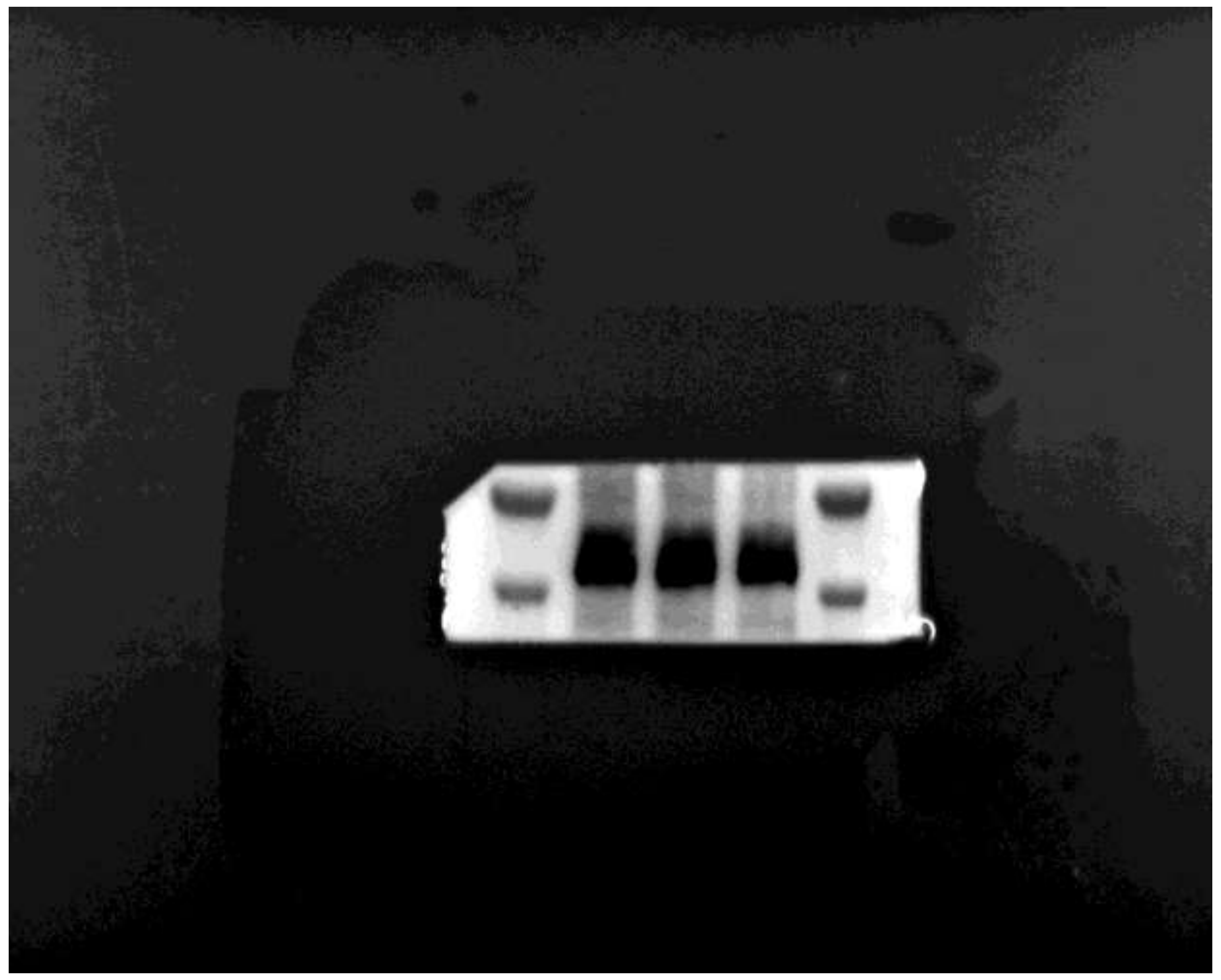

β-actin
